# Application of a General Computer Algorithm Based on the Group-Additivity Method for the Calculation of Two Molecular Descriptors at Both Ends of Dilution: Liquid Viscosity and Activity Coefficient in Water at Infinite Dilution

**DOI:** 10.3390/molecules23010005

**Published:** 2017-12-21

**Authors:** Rudolf Naef, William E. Acree

**Affiliations:** 1 Department of Chemistry, University of Basel, 4003 Basel, Switzerland; 2 Department of Chemistry, University of North Texas, Denton, TX 76203, USA; acree@unt.edu

**Keywords:** liquid viscosity, activity coefficient at infinite dilution, group-additivity method

## Abstract

The application of a commonly used computer algorithm based on the group-additivity method for the calculation of the liquid viscosity coefficient at 293.15 K and the activity coefficient at infinite dilution in water at 298.15 K of organic molecules is presented. The method is based on the complete breakdown of the molecules into their constituting atoms, further subdividing them by their immediate neighborhood. A fast Gauss–Seidel fitting method using experimental data from literature is applied for the calculation of the atom groups’ contributions. Plausibility tests have been carried out on each of the calculations using a ten-fold cross-validation procedure which confirms the excellent predictive quality of the method. The goodness of fit (Q^2^) and the standard deviation (σ) of the cross-validation calculations for the viscosity coefficient, expressed as log(η), was 0.9728 and 0.11, respectively, for 413 test molecules, and for the activity coefficient log(γ)^∞^ the corresponding values were 0.9736 and 0.31, respectively, for 621 test compounds. The present approach has proven its versatility in that it enabled the simultaneous evaluation of the liquid viscosity of normal organic compounds as well as of ionic liquids.

## 1. Introduction

In recent years, among the many computational methods for the prediction of physico-chemical properties of organic compounds, such as those derived from (quantum-)theoretical considerations, multiple linear regression approaches based on correlations between further properties of interest, cluster analysis, principal component analysis or group-additivity methods, the latter method has gained increasing interest due to its wide-ranging applicability for the evaluation of numerous molecular descriptors. Recently, two papers [1,2] demonstrated its versatility in that a single computer algorithm using a radical form of the atom-groups additivity method was able to reliably predict ten molecular descriptors: heats of combustion, solvation, sublimation and vaporization, entropy of fusion, partition coefficient logP_o/w_, solubility logS_water_, refractivity, polarizability and toxicity. The availability of the experimental values of the liquid viscosity coefficient (η) and the activity coefficient at infinite dilution in water log(γ)^∞^ of several hundred organic compounds from various literature references gave reason to try to extend the atom-groups additivity approach described in [1] to these two descriptors, which coincidentally are both at the extreme ends of dilution.

The viscosity is an important property of liquid compounds, its knowledge required in particular in the transport business of bulk quantities of liquids as well as in the field of ionic liquids. Earlier attempts to predict the liquid viscosity coefficient of organic compounds have been developed on a statistical mechanics model based on the square well intermolecular potential [3], or have been carried out applying multiple linear regression and artificial neural network modelling methods using a limited number of descriptors as input [4,5], or are based on a quantitative structure-property relationship (QSPR) approach using a five-descriptor equation [6], or use a combination of partial least-square and QSPR technique starting with 18 mostly experimental parameters, finally ending with a model with nine descriptors [7].

Knowledge of the activity coefficient of a molecule at infinite dilution γ^∞^ is important, e.g., to characterize liquid mixtures, to screen solvents for extractive distillation processes or to predict the existence of azeotropes. Several methods for the γ^∞^ calculation have been published, based on QSPR or group contribution methods (e.g., ASOG or UNIFAC), summed up in [8]. An interesting approach founded on the ant-colony optimization (ACO) method, which allowed to select five relevant descriptors out of 1160 quantum-chemical and topological descriptors [9].

All the mentioned prediction methods rely on various series of either experimental or theoretically evaluated descriptors of the compounds. The advantage of the present method lies in the fact that, on the one hand, a unified computer algorithm enabled the evaluation of the group parameters for both descriptors from experimental data, and, on the other hand, that for their subsequent predictive calculation even a 2D sketch of a moleculeon a sheet of paper would be of sufficient help.

## 2. General Procedure

The present compounds with known viscosity or activity coefficient values are stored as 3D-geometry-optimized structures in a knowledge database encompassing at present more than 30,000 records covering the fields of pharmaceuticals, plant protection, dyes, ionic liquids, liquid crystals, metal-organics, lab intermediates and many more, and containing a large number of experimental and calculated molecular descriptors.

The atom-groups additivity method underlying the present algorithm for the calculation of the two title descriptors has been detailed in an earlier publication [1]. Accordingly, the definition and meaning of the atom groups in the respective parameters tables for the two descriptors remain identical and are explained in Table 1 of [1] and its footnotes. (For better readability of a neighbors term containing iodine its symbol is written as J.) In order to include the ionic liquids a number of further atom groups representing their charged moieties had to be included (see Table 1), which are treated the same way by the computer algorithm as the remaining ones.

**Figure 1:**
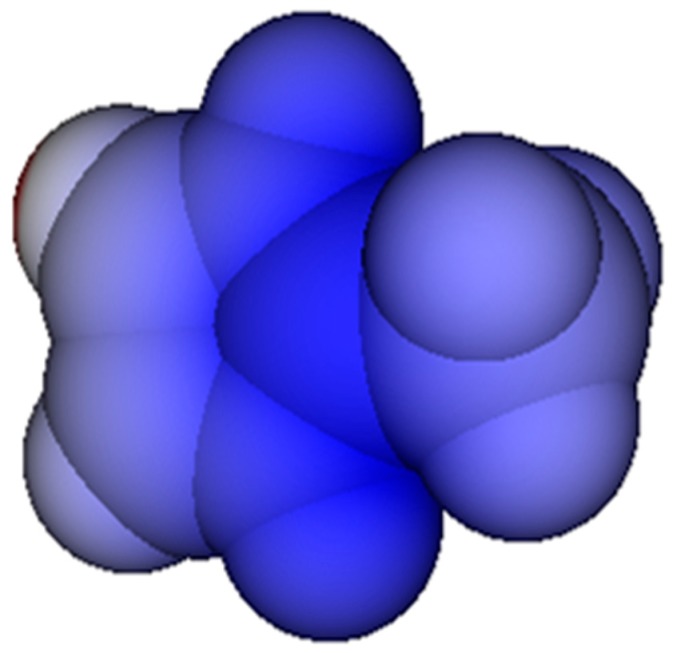
Charge distribution on the VdW surface of the 2-methylimidazolium ion. Positive charge intensity indicated by depth of blue color. (EHMO calculation and graphics by ChemBrain IXL).

While most of the group definitions are self-explanatory, group No. 3 requires some additional explanation: in drawings of compounds such as imidazolium (or guanidinium, for that matter) the positive charge is usually assumed to be localized on one of the nitrogen atoms, which inherently implies an asymmetrical charge distribution in these molecules where there is none. This creates an ambiguity problem in truly asymmetrical cases where one or more of these nitrogen atoms carry additional, different substituents: on which nitrogen atom should the positive charge now be positioned? The best answer is given by quantum-theoretical calculations, e.g., by the extended Hückel MO (EHMO) method [10], which prove that the positive charge is indeed essentially centered on the carbon atom between the nitrogen atoms (see Figure 1)! This is also true for analogous compounds carrying alkyl substituents at the nitrogen atoms (which would be represented by the atom group No. 4 in Table 1).

Accordingly, the representation of e.g., the 2-methylimidazolium ion applied to the present group-additivity calculations has the positive charge assigned to the carbon atom at position 2, which on the other hand is bound to the two neighbor nitrogens by aromatic bonds. (Analogously, the positive charge of the guanidinium ion would be assigned to the central carbon atom, which is bound to each of the three nitrogen atoms by aromatic bonds).

Following the calculation procedure described in [1], the computer algorithm breaks down the molecule to be evaluated into its constituting atom groups and checks for their occurrence in the respective group-parameters table generated earlier. In order to be eligible for the molecule’s descriptor evaluation, the algorithm ensures that not only each of the molecule’s atom groups is found in the group-parameters table but also that each of the groups found is “valid”, i.e., that each has been represented in the preceding parameters-evaluation process by at least three independent molecules with known experimental descriptor value. On condition that these two requirements are fulfilled, the descriptor calculation follows the general Equation (1), where *Y* is the descriptor, *a_i_* and *b_j_* are the contributions, *A_i_* is the number of occurrences of the *i*th atom group, and *B_j_* is the number of occurrences of the *j*th special group and *C* is a constant:

(1) $$Y=\underset{i}{\sum }a_{i}A_{i}+\underset{j}{\sum }b_{j}B_{j}+C$$

For each of the presented two descriptors a separate group-parameters table has been prepared. The evaluation of the group contributions according to the detailed description in [1] was immediately followed by a plausibility test based on a ten-fold cross-validation procedure, wherein it was ensured that each of the compounds has been introduced alternatively as both a test or training sample. In row A to H at the end of each parameters table the results are collected. The correlation diagrams and histograms in the respective sections below show the results of the training and cross-validation calculations in black and red colors, respectively.

In the calculation processes of the two group-parameters tables it turned out that for an optimal viscosity-coefficient prediction the second summand in Equation (1) was not needed as there was no special group required, whereas for the prediction of the activity coefficient log(γ)^∞^ the best value for the constant C was zero.

Looking at the rightmost column of the group-parameters tables showing the number of molecules representing a given atom group, one may notice that some of the atom groups are represented by less than three molecules. These atom groups are therefore not applicable for descriptor predictions; nevertheless, they have been left in the parameters tables for potential future use in this continuous project. As the parameters tables show, calculations have been restricted to molecules containing the elements H, B, C, N, O, P, S, Si and/or halogen.

## 3. Results

### 3.1. General Remarks

(1) Cross-validation data in the following figures are superpositioned in red.
(2) Generally, compounds, the experimental values of which exceeded by more than three times the cross-validated standard error, have been excluded from group-parameters calculations and have been collected in a list of outliers.
(3) Lists of molecules used in these studies are available as standard SDF files, stored in the Supplementary Materials, which also encompasses the lists of results with molecule names, experimental, training and cross-validation values and, additionally, lists of experimental outliers.

### 3.2. Liquid Viscosity Coefficient

Conventionally, the standard temperature for the viscosity values has been chosen to be 293.15 K in order to compare them with that of water, which then conveniently is 1 centipoise (1.0087 cP, to be precise). Accordingly, only viscosity coefficients have been considered in the literature which have been measured or reduced to this temperature. In the present study the viscosity coefficients have been transformed into their decimal logarithm and entered into the group-additivity calculation as log(η). The main sources of experimental viscosity data have been the collective papers of Suzuki et al. [5,7] and Katritzky et al. [6], supplemented by more recently published experimental results for alkanes [11,12,13,14], haloalkanes [15,16], alkanols [17,18,19,20], alkylamines [21,22,23,24], aminoalcohols [25,26,27], ethers [28,29], aminoethers [30], acetals [31], ketones [32], esters [33,34,35,36,37,38,39,40,41,42,43], hydroxyesters [44,45], carbonate esters [46], and amides [47,48,49,50,51,52]. Beyond these, experimental data have been added for compounds with atom groups that have not yet been represented in the parameters table: phosphoric acid esters [53,54,55], phosphoric acid amides [56], siloxanes [57] and in particular ionic liquids [32,58,59,60,61,62,63,64,65,66,67,68,69,70,71,72]. Table 2 lists the final result of the atom-groups parameters calculation, based on 501 compounds. Attempts to further improve the result by the inclusion of certain special groups described in Table 2 of paper [1], such as correction factors for pure hydrocarbons or for methylene chains, unanimously yielded slightly lower correlation coefficients and higher standard deviations.

Entries A to H at the bottom of Table 1 show that 126 atom groups were required in the atom-groups parameters calculation to comprise 501 compounds with known experimental viscosity data, of which 76 atom groups were finally “valid”, i.e., reliable for viscosity-coefficient predictions. Accordingly, only 460 compounds of the entire training set and 413 of the ten cross-validation test sets were fit for prediction.

**Figure 2:**
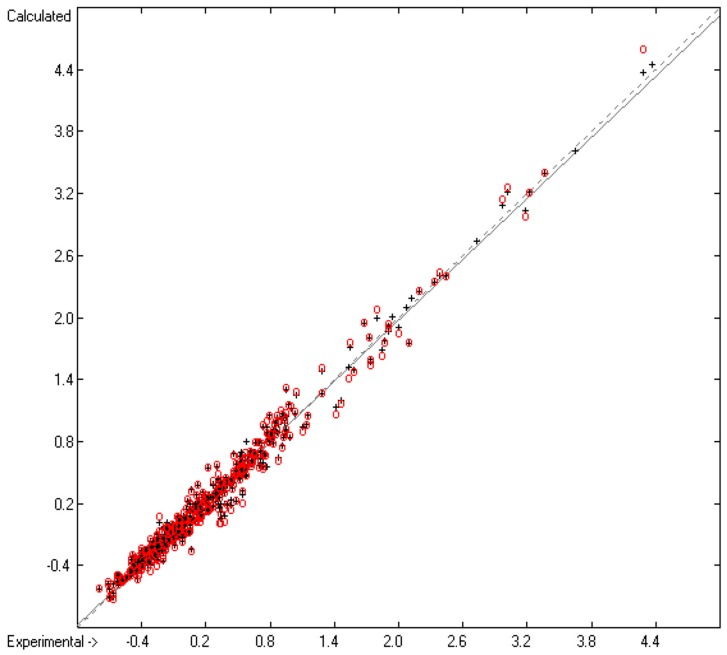
Correlation Diagram of the Viscosity Coefficients (N = 460; R^2^ = 0.9831; Q^2^ = 0.975; regression line: intercept = 0.0137; slope = 0.9824).

**Figure 3:**
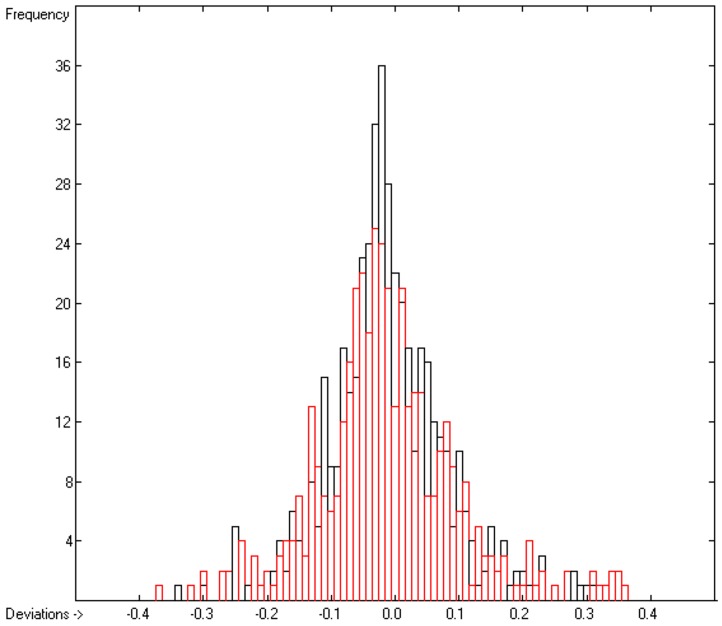
Histogram of the Viscosity Coefficients (S = 0.11; Exp. Values range from −0.785 to +4.3732).

The correlation diagram in Figure 2 reveals a very good compliance between the training and cross-validation results, confirmed by the close similarity of standard deviations R^2^ and Q^2^ (lines B and F in Table 2). The corresponding histogram in Figure 3 exhibits a slightly distorted Gaussian bell curve, the maximum of which being shifted by 0.02 to the negative deviations (indicating smaller experimental values than predicted), which might be ascribed to the relatively small number of experimental data.

Of particular interest is the question as to how well the prediction of the viscosity of ionic liquids performs. For 15 of the presently 33 ionic liquids, for which experimental data were available, predictions were possible. Their log(η) ranged between 1.951 and 4.3732; hence, in Figure 2 they are all positioned at the upper half of the correlation diagram. Evidently, their data points are in excellent conformance with those of the “normal” compounds, which may be surprising considering the additional interactive forces acting between their ionic moieties, but these extra effects are inherently considered in the assigned atom-groups parameters listed in Table 1. Nevertheless, five out of the 33 ionic liquids had to be removed from calculations as their deviation exceeded prediction by far more than three times the cross-validated standard deviation. They are collected in the list of outliers, available in the Supplementary Materials.

How do these results compare with the prediction methods published earlier? Quantitative structure-activity relationship (QSAR) techniques, described in [7], applied on a set of 237 compounds and using 18 physical properties as input into multiple linear as well as partial least squares regression calculations, yielded correlation coefficients of 0.933 and 0.931, respectively, and corresponding standard errors of 0.144 and 0.146. Later, a quantitative structure-property relationship (QSPR) study [6], founded on 361 compounds and using five molecular structural descriptors including electrostatic and quantum chemical properties, resulted in a correlation coefficient of 0.854 and a standard error of 0.22. The multiple linear regression and artificial neural network (ANN) back-propagation methods, outlined in [4], based on 361 compounds and nine physical and structural descriptors, yielded a correlation coefficient of 0.92 and 0.93, respectively, and corresponding standard errors of 0.17 and 0.16 units. In a later paper [5], the same authors presented slightly better results with a set of 440 compounds, using the same ANN approach and input descriptors, which produced correlation coefficients for the training, validation and test sets of 0.956, 0.932 and 0.884, respectively, with corresponding standard errors of 0.122, 0.134 and 0.148 units. Evidently, comparing these results with the data collected at the bottom of Table 2, none of the cited prediction methods achieved the accuracy of the present approach and, beyond this, the present method even allows a reliable prediction of the viscosity coefficient at 20 °C simply by hand, using paper and pencil, Table 2 and Equation (1). The only drawback is the condition that each atom group in a given molecule must be found in the table and that it is preferably represented by three or more molecules (shown in the rightmost column). A scan of the database of currently 30,125 compounds, which can be viewed as representative for the entire structural coverage of chemicals, reveals that at present this is the case for about 39% of all compounds, due to the relatively small experimental basis of only 501 compounds.

### 3.3. Activity Coefficient at Infinite Solution in Water

Generally, the activity coefficient γ^∞^ has been published in its logartithmic form log(γ)^∞^ and has been measured at 298.15 K. In some cases, where γ^∞^ itself or its logarithmus naturalis was cited, the data have been translated into their decimal logarithm. In addition, only values have been considered which have been measured at or reduced to 298.15 K. Primary sources of experimental data have been the collective reports mentioned earlier [8,9]. Additional data have been found for 1-propoxypropan-2-ol [73], several alkyl and alkenyl alcohols and alkylbenzenes [74], valeric and crotonic aldehyde [75], variously substituted benzoic acids [76,77], naphthoic acids [78,79], isatin [80], 2-cyanoguanidine [81], florfenicol [82], thiamphenicol [83] and various sulfonamides [84,85]. In total, the number of compounds with experimental log(γ)^∞^ data amounted to 709, of which 34 turned out to be outliers (a list of them is available in the Supplementary Materials), as their experimental values differed by more than three times the cross-validated standard error from prediction. The remaining 675 compounds represented 113 atom groups, of which 75 have been defined as valid for predictions (see line A of Table 3). A number of calculations, which tentatively in- or excluded certain special groups, revealed that consideration of alkanes and unsaturated hydrocarbons (special groups 115 and 116 in Table 3) as separate entities significantly improved the values of the correlation coefficient R^2^ (from 0.9621 to 0.9788) as well as the corresponding standard error (from 0.37 to 0.27), whereas the inclusion of intramolecular hydrogen bonds (special group 114) only had a minor effect, probably due to the small number of only six examples. Nevertheless, in view of future data input this latter group has been left in the parameters table.

**Figure 4:**
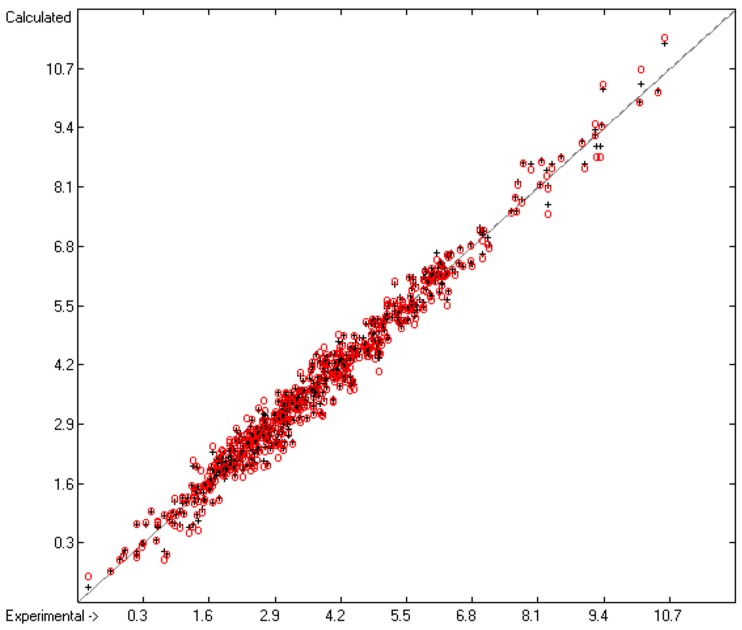
Correlation Diagram of the log(γ)^∞^ Data (N = 634; R^2^ = 0.9789; Q^2^ = 0.9737; regression line: intercept = 0.0022; slope = 0.9972).

**Figure 5:**
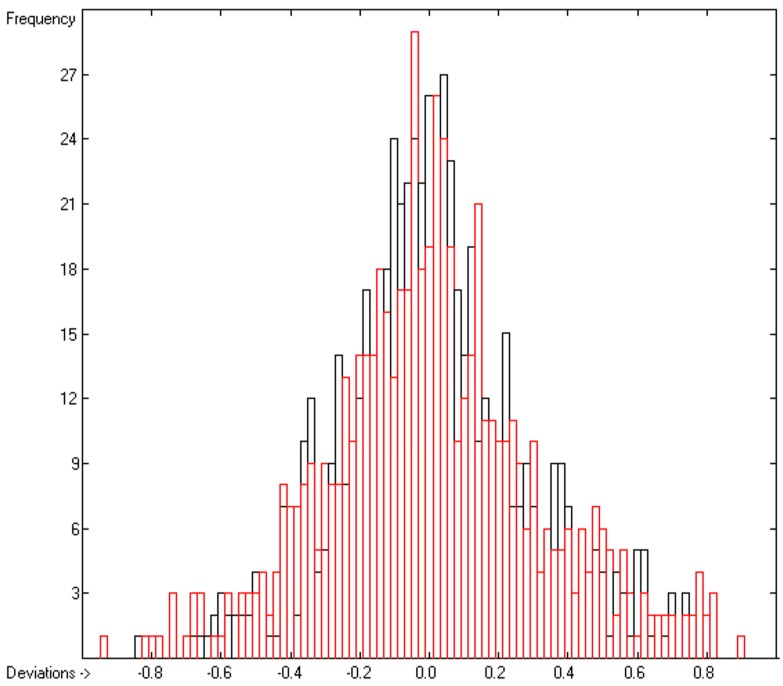
Histogram of the log(γ)^∞^ Data (S = 0.31; Exp. Values range from −0.762 to +10.624).

The correlation diagram in Figure 4 shows a very good conformance between the training and cross-validation test values, which is reflected in the very similar values of R^2^ and Q^2^. The intercept and slope of the regression line confirm that in this case a constant C is not required in the prediction calculations pursuant to Equation (1). Due to the fairly limited number of samples, on the other hand, the histogram in Figure 5 does not exhibit a perfect Gaussian bell curve but at least its maximum is reasonably well centred at the zero deviation point.

Comparison of the present result with those published in earlier articles [8,9] reveals that it lies in the same range of prediction accuracy: Abraham’s method, described in [8], being based on the five descriptors: excess molar refractivity, dipolarity/polarizability, overall or summation hydrogen bond acidity and basicity, and the McGowan volume, yielded a correlation coefficient R^2^ of 0.977 and a leave-one-out cross-validation correlation coefficient Q^2^ of 0.976 and corresponding standard errors of 0.284 and 0.29, respectively, for 655 structurally diverse compounds; the ant-colony optimization method, outlined in [9], limited to 105 hydrocarbons and founded on four topological descriptors and the refractivity, resulted in a correlation coefficient R^2^ of 0.9893 and a standard error of 0.3996 for the calibration set, and a Q^2^ of 0.9891 and a standard error of 0.3865 for the prediction set. The main advantage of the present method lies in its ease of use in that—just like in the previous subsection—a simple 2D drawing is needed to help to find all the compound’s atom groups and then sum up their contributions according to Table 3. In addition, for hydrocarbons, each carbon atom would contribute according to entry 115 or 116 in Table 3. The only disadvantage of the present approach lies in its limited range of molecules for which log(γ)^∞^ is calculable, due to the relatively small amount of “valid” atom groups as a result of the limited number of experimental data—a weakness, however, which is gradually being remedied by means of the input of further experimental data in this ongoing project. At present, for 51% of the compounds of the current database the log(γ)^∞^ value has been evaluated.

## 4. Conclusions

Ease of use and reliability of the predictions was the goal of the presented subject. While the former was in the hands of the method developer, the latter highly depended on the experimental data provided by the countless scientific publishers. The present results, together with those outlined in the previous publications [1,2], prove the enormous versatility of the atom-groups additivity method, particularly on applying the radical breakdown of the molecules as described, in that, including the present ones, the following 13 molecular descriptors can be calculated at once (some of them indirectly) in a split second on a desktop computer: the heats of combustion, formation, solvation, sublimation and vaporization, the entropy of fusion, the partition coefficient logP_o/w_, the solubility logS_water_, the refractivity, the polarizability, the toxicity against the protozoan *Tetrahymena pyriformis* and, as has been demonstrated here, the viscosity coefficient log(η) and the activity coefficient log(γ)^∞^. The disadvantage of the radical breakdown of the molecules which inevitably leads to a large number of particularized atom groups and thus excludes molecules from any calculation for which not all of their atom groups have a defined contribution, is well compensated on the one hand by the accuracy of prediction for those compounds for which calculation is possible, in most cases even by the simple paper-and-pencil approach for finding the atom groups in a given molecule and summing up their contributions, and on the other hand by the enablement of a standardized computer algorithm, allowing a simple extension of each of the atom-groups parameters lists at the input of any further, future experimental data, which again would extend the scope of calculable molecular structures. The reliability of the predictions, however, only increases with the accuracy of any future input. The present work is part of an ongoing project called ChemBrain IXL available from Neuronix Software (www.neuronix.ch, Rudolf Naef, Lupsingen, Switzerland).

## Figures and Tables

**Table 1:** Atom-Group Examples for Ionic Liquids and their Meaning.

No.	Atom Type	Neighbours	Meaning	Example
1	B(−)	F4	BF_4_^−^	tetrafluoroborate
2	C aromatic	H:C:N(+)	C:CH:N^+^	C2 in pyridinium
3	C(+) aromatic	C:N2	N:C^+^(C):N	C2 in 2-methylimidazolium
4	N aromatic	C2:C(+)	C-N(C):C^+^	N1 in 1-methylimidazolium
5	N(+) aromatic	C:C2	C:N^+^(C):C	N in 1-methylpyridinium
6	N(−)	S2	S-N^−^-S	bis(trifluoromethanesulfonyl)amide
7	P(+)	C4	PC_4_^+^	tetramethylphosphonium
8	P(−)	F6	PF_6_^−^	hexafluorophosphate
9	S4	CO=O2(−)	C-SO3^−^	methylsulfonate

**Table 2:** Atom groups and their contributions for liquid viscosity-coefficient calculations.

Entry	Atom Type	Neighbours	Contribution	Occurrences	Molecules
1	Const		−0.70	501	501
2	B(−)	F4	1.50	4	4
3	C sp3	H3C	−0.06	694	389
4	C sp3	H3C(+)	2.07	3	3
5	C sp3	H3N	0.54	31	21
6	C sp3	H3N(+)	0.69	2	2
7	C sp3	H3O	0.43	31	25
8	C sp3	H3S	0.19	7	5
9	C sp3	H3Si	0.13	18	2
10	C sp3	H2C2	0.09	1634	313
11	C sp3	H2CN	0.63	87	51
12	C sp3	H2CN(+)	1.75	13	12
13	C sp3	H2CO	0.51	182	118
14	C sp3	H2CP(+)	0.43	12	3
15	C sp3	H2CS	0.36	20	15
16	C sp3	H2CCl	0.33	23	20
17	C sp3	H2CBr	0.44	14	12
18	C sp3	H2CJ	0.59	3	3
19	C sp3	H2O2	0.93	1	1
20	C sp3	HC3	0.21	118	92
21	C sp3	HC2N	0.65	8	7
22	C sp3	HC2N(+)	0.89	1	1
23	C sp3	HC2O	0.69	17	16
24	C sp3	H2CP	0.08	2	1
25	C sp3	HC2S	0.50	4	4
26	C sp3	HC2Cl	0.41	5	5
27	C sp3	HC2Br	0.58	1	1
28	C sp3	HC2J	0.67	1	1
29	C sp3	HCO2	1.11	3	1
30	C sp3	HCF2	1.49	1	1
31	C sp3	HCCl2	0.37	3	3
32	C sp3	HCBr2	0.85	2	1
33	C sp3	C4	0.44	14	10
34	C sp3	C3O	0.89	6	6
35	C sp3	C3S	0.72	3	3
36	C sp3	C3Cl	0.60	1	1
37	C sp3	C3J	0.83	1	1
38	C sp3	C2O2	1.14	1	1
39	C sp3	CSF2	0.00	1	1
40	C sp3	CPF2(−)	0.19	6	2
41	C sp3	CF3	−0.15	10	6
42	C sp3	CF2Cl	0.55	1	1
43	C sp3	CFCl2	0.00	1	1
44	C sp3	CCl3	0.72	1	1
45	C sp3	SF3	0.43	14	7
46	C sp2	H2=C	−0.06	42	40
47	C sp2	HC=C	0.05	74	52
48	C sp2	HC=O	0.26	7	7
49	C sp2	H=CN	0.48	28	14
50	C sp2	H=CO	−0.01	6	5
51	C sp2	H=CS	0.26	5	3
52	C sp2	H=CCl	0.17	5	3
53	C sp2	HN=O	0.51	3	3
54	C sp2	HO=O	0.12	9	9
55	C sp2	C2=C	0.13	11	11
56	C sp2	C2=O	0.37	15	14
57	C sp2	C=CS	0.35	1	1
58	C sp2	CN=O	0.66	4	4
59	C sp2	CN=O(+)	−3.99	1	1
60	C sp2	CO=O	0.26	100	83
61	C sp2	CO=O(−)	0.95	3	3
62	C sp2	C=OBr	0.44	1	1
63	C sp2	=CCl2	0.32	4	3
64	C sp2	O2=O	0.29	3	3
65	C aromatic	H:C2	0.07	441	97
66	C aromatic	H:C:N	0.24	7	4
67	C aromatic	H:C:N(+)	0.00	18	9
68	C aromatic	:C3	0.31	4	2
69	C aromatic	C:C2	0.20	90	73
70	C aromatic	C:C:N	0.40	3	2
71	C aromatic	:C2N	0.28	9	9
72	C aromatic	:C2N(+)	0.83	3	3
73	C aromatic	:C2O	0.22	9	6
74	C aromatic	:C2S	1.42	3	3
75	C aromatic	:C2F	0.09	4	4
76	C aromatic	:C2Cl	0.25	6	4
77	C aromatic	:C2Br	0.36	2	2
78	C aromatic	:C2J	0.57	1	1
79	C(+) aromatic	H:N2	0.40	10	10
80	C(+) aromatic	C:N2	−3.06	3	3
81	C sp	H#C	−0.17	1	1
82	C sp	C#C	0.00	1	1
83	C sp	C#N	0.35	19	19
84	C sp	N#N(−)	−0.02	2	1
85	C sp	#NS(−)	1.59	1	1
86	N sp3	H2C	−0.21	19	18
87	N sp3	H2C(pi)	0.66	7	7
88	N sp3	HC2	−0.74	12	12
89	N sp3	HC2(pi)	0.02	3	3
90	N sp3	HC2(2pi)	−0.23	1	1
91	N sp3	C3	−1.38	12	12
92	N sp3	C3(pi)	−0.92	6	6
93	N sp3	C2P	−0.66	3	1
94	N(+) sp3	H3C	0.14	2	2
95	N(+) sp3	C4	−0.95	1	1
96	N aromatic	:C2	−0.12	5	5
97	N aromatic	C2:C(+)	−0.05	26	13
98	N(+) aromatic	C:C2	−0.54	9	9
99	N(+) sp2	CO=O(−)	−0.18	5	5
100	N(+) sp2	O2=O(−)	0.74	1	1
101	N(−)	C2	0.00	1	1
102	N(−)	S2	0.86	7	7
103	O	HC	0.58	58	45
104	O	HC(pi)	0.63	18	18
105	O	C2	−0.79	40	31
106	O	C2(pi)	−0.25	97	80
107	O	C2(2pi)	0.20	6	6
108	O	CP	−0.13	9	3
109	O	CP(pi)	0.29	3	1
110	O	CS	−0.06	2	2
111	O	Si2	0.00	9	2
112	P4	C2O=O(−)	−0.88	1	1
113	P4	N3=O	0.00	1	1
114	P4	O3=O	0.00	4	4
115	P(+)	C4	−0.22	3	3
116	P(−)	F6	0.84	2	2
117	P(−)	C3F3	−0.07	2	2
118	S2	HC	−0.09	13	13
119	S2	HC(pi)	−0.98	1	1
120	S2	C2	−0.21	9	9
121	S2	C2(2pi)	−0.10	3	3
122	S4	C2=O	0.67	1	1
123	S4	CN=O2(−)	0.00	14	7
124	S4	CO=O2(−)	−1.08	4	4
125	S4	O2=O2(−)	0.00	2	2
126	Si	C2O2	0.00	9	2
A	Based on	Valid groups	76		501
B	Goodness of fit	R^2^	0.9831		460
C	Deviation	Average	0.07		460
D	Deviation	Standard	0.10		460
E	K-fold cv	K	10		413
F	Goodness of fit	Q^2^	0.975		413
G	Deviation	Average (cv)	0.08		413
H	Deviation	Standard (cv)	0.11		413

**Table 3:** Atom groups and their contributions for log(γ)^∞^ calculations.

Entry	Atom Type	Neighbours	Contribution	Occurrences	Molecules
1	C sp3	H3C	0.99	776	422
2	C sp3	H3N	0.91	27	20
3	C sp3	H3N(+)	0.38	1	1
4	C sp3	H3O	0.86	50	45
5	C sp3	H3S	1.2	9	6
6	C sp3	H2C2	0.6	972	284
7	C sp3	H2CN	0.27	52	29
8	C sp3	H2CN(+)	0.8	3	3
9	C sp3	H2CO	0.21	131	101
10	C sp3	H2CS	0.19	9	6
11	C sp3	H2CF	0.7	1	1
12	C sp3	H2CCl	1.41	23	19
13	C sp3	H2CBr	1.81	15	13
14	C sp3	H2CJ	2.45	5	5
15	C sp3	HC3	0.14	96	71
16	C sp3	HC2N	0.28	6	6
17	C sp3	HC2N(+)	0.37	1	1
18	C sp3	HC2O	−0.39	52	49
19	C sp3	HC2S	−0.14	3	2
20	C sp3	HC2Cl	1.02	4	4
21	C sp3	HC2Br	1.25	3	3
22	C sp3	HC2J	1.85	1	1
23	C sp3	HCCl2	1.79	7	6
24	C sp3	HCBr2	2.23	2	1
25	C sp3	C4	−0.46	37	33
26	C sp3	C3O	−1.14	21	20
27	C sp3	C3F	1.29	1	1
28	C sp3	C2F2	1.12	18	4
29	C sp3	CF3	1.82	10	6
30	C sp3	CF2Cl	2.43	4	3
31	C sp3	CFCl2	2.19	1	1
32	C sp3	CCl3	2.76	5	4
33	C sp2	H2=C	0.98	54	45
34	C sp2	HC=C	0.6	109	69
35	C sp2	HC=O	−0.3	17	17
36	C sp2	H=CN	0.9	6	4
37	C sp2	H=CO	0.88	8	6
38	C sp2	H=CS	−0.99	3	3
39	C sp2	H=CCl	1.54	7	5
40	C sp2	HN=O	−0.7	2	2
41	C sp2	HO=O	0.84	8	8
42	C sp2	C2=C	0.24	15	15
43	C sp2	C2=N	1.59	2	2
44	C sp2	C=CN	−2.47	1	1
45	C sp2	C2=O	−1.17	38	35
46	C sp2	C=CO	0.54	7	5
47	C sp2	C=CS	0.09	1	1
48	C sp2	CN=O	−0.23	34	25
49	C sp2	CO=O	0.06	91	83
50	C sp2	=CF2	1.51	2	1
51	C sp2	=CCl2	2.3	3	2
52	C sp2	N2=N	0.4	1	1
53	C sp2	N2=O	0.41	15	15
54	C sp2	N=NS	−0.04	2	2
55	C sp2	O2=O	0.88	2	2
56	C aromatic	H:C2	0.56	1318	270
57	C aromatic	H:C:N	−0.39	25	17
58	C aromatic	:C3	0.16	92	27
59	C aromatic	C:C2	0.06	209	138
60	C aromatic	C:C:N	−1.04	10	8
61	C aromatic	:C2N	−0.65	90	65
62	C aromatic	:C2N(+)	0.56	43	33
63	C aromatic	:C2O	0.13	67	58
64	C aromatic	:C2S	0.34	42	40
65	C aromatic	:C2F	0.72	22	8
66	C aromatic	:C2Cl	1.26	108	59
67	C aromatic	:C2Br	1.5	30	16
68	C aromatic	:C2J	1.88	6	5
69	C aromatic	:CN:N	−0.66	3	3
70	C aromatic	:C:NCl	1.22	2	2
71	C aromatic	N:N2	0.14	4	3
72	C aromatic	:N2Cl	−0.74	1	1
73	C sp	H#C	0.69	13	10
74	C sp	C#C	0.22	11	9
75	C sp	C#N	−0.07	10	10
76	C sp	N#N	0	1	1
77	C sp	=N=S	3.15	1	1
78	N sp3	H2C	−1.57	10	10
79	N sp3	H2C(pi)	0.16	38	37
80	N sp3	HC2	−1.62	6	6
81	N sp3	HC2(pi)	−0.11	6	5
82	N sp3	HC2(2pi)	−0.95	37	28
83	N sp3	HCS	−0.59	1	1
84	N sp3	HCS(pi)	−1.13	32	32
85	N sp3	C3	−1.62	10	9
86	N sp3	C3(pi)	−1.48	6	6
87	N sp3	C3(2pi)	−1.59	4	4
88	N sp3	C2N(pi)	−1.91	1	1
89	N sp3	C2N(2pi)	0	1	1
90	N sp3	C2O(pi)	−0.33	2	2
91	N sp3	C2S	−0.97	2	2
92	N sp3	C2S(2pi)	−2.06	1	1
93	N sp2	H=C	0.79	1	1
94	N sp2	C=C	−1.63	5	5
95	N sp2	C=N	0.21	2	1
96	N aromatic	:C2	0.31	24	20
97	N aromatic	:C:N	−0.16	2	1
98	N(+) sp2	CO=O(−)	0.12	48	38
99	O	HC	−0.84	81	77
100	O	HC(pi)	−0.9	67	63
101	O	HO	−0.39	3	2
102	O	C2	−0.37	36	34
103	O	C2(pi)	−0.41	96	84
104	O	C2(2pi)	−0.61	11	11
105	O	CN	0	2	2
106	O	CO	−0.15	3	2
107	S2	HC	1.12	7	6
108	S2	C2	0.29	3	3
109	S2	C2(2pi)	2.31	5	5
110	S2	CS	0.42	2	1
111	S4	C2=O	−4.04	2	2
112	S4	C2=O2	−1.81	2	2
113	S4	CN=O2	−0.07	36	36
114	H	H Acceptor	0.14	6	6
115	Alkane	No of C atoms	0.19	272	39
116	Unsaturated HC	No of C atoms	0.03	844	92
A	Based on	Valid groups	75		675
B	Goodness of fit	R^2^	0.9789		634
C	Deviation	Average	0.21		634
D	Deviation	Standard	0.27		634
E	K-fold cv	K	10		616
F	Goodness of fit	Q^2^	0.9737		616
G	Deviation	Average (cv)	0.23		616
H	Deviation	Standard (cv)	0.31		616

## Supplementary Materials

The following files are available online. The list of compounds, their experimental and calculated data and 3D structures of the viscosity-coefficient calculations are available under the names of “S1. Experimental and Calculated Viscosity-Data Table.doc” and “S2. Compounds List of Viscosity Calculations.sdf”. A list of their outliers has been added under the name of “S3. Compounds List of Viscosity Outliers.xls”. The set of experimental and calculated data of activity coefficients calculations is available under the name of “S4. Experimental and Calculated Activity-Coefficient-Data Table.doc”, the corresponding list of compounds under the name of “S5. Compounds List of Activity-Coefficient Calculations.sdf” and the respective outliers list under the name of “S6. Compounds List of Activity-Coefficient Outliers.xls”. The figures are available as tif files and the tables as doc files under the names given in the text.
