# Supplementary material for: Application of a General Computer Algorithm Based on the Group-Additivity Method for the Calculation of Two Molecular Descriptors at Both Ends of Dilution: Liquid Viscosity and Activity Coefficient in Water at Infinite Dilution
Source: Molecules. 2017 Dec 21;23(1):5. doi: 10.3390/molecules23010005 (PMC5943952; doi:10.3390/molecules23010005)
Supplement: Supplementary file 1 [file molecules-23-00005-s001.zip › S1. Experimental and Calculated Viscosity-Data Table.doc]

|  | **Molecules sorted by deviation** |  |  |  |  |
| --- | --- | --- | --- | --- | --- |
| **Entry** | **Molecule name** | **log (η) exp** | **log (η) calc** | **log (η) test** | **Deviation** |
| 1 | Isoamyl acetate | -0.06 | -0.06 | -0.06 | 0.00 |
| 2 | Butyronitrile | -0.21 | -0.23 | -0.21 | 0.00 |
| 3 | Benzyl methacrylate | 0.41 | 0.38 | 0.41 | 0.00 |
| 4 | 1-Butyl-3-methylimidazolium acetate | 2.74 | 2.74 |  | 0.00 |
| 5 | Undecylcyclohexane | 0.80 | 0.80 | 0.80 | 0.00 |
| 6 | Allyl cyanide | -0.29 | -0.27 | -0.29 | 0.00 |
| 7 | Thiophene | -0.18 | -0.18 |  | 0.00 |
| 8 | Ethyl-n-propylether | -0.49 | -0.50 | -0.49 | 0.00 |
| 9 | 1-Bromononane | 0.30 | 0.31 | 0.30 | 0.00 |
| 10 | Isopropyl methyl sulfide | -0.34 | -0.34 |  | 0.00 |
| 11 | Diethyl malonate | 0.33 | 0.31 | 0.33 | 0.00 |
| 12 | Neohexane | -0.44 | -0.41 | -0.44 | 0.00 |
| 13 | Chlorobenzene | -0.10 | -0.10 |  | 0.00 |
| 14 | Octylbenzene | 0.41 | 0.42 | 0.41 | 0.00 |
| 15 | Methyl isobutyl ketone | -0.23 | -0.21 | -0.23 | 0.00 |
| 16 | 3-Nitrotoluene | 0.37 | 0.37 |  | 0.00 |
| 17 | 1,4-Dichlorobutane | 0.13 | 0.14 | 0.13 | 0.00 |
| 18 | Benzylmethylamine | 0.25 | 0.28 | 0.25 | 0.00 |
| 19 | 1-Propyl-2,3-dimethylimidazolium bis(trifluoromethanesulfonyl) amide | 2.09 | 2.09 |  | 0.00 |
| 20 | 2-Nitrotoluene | 0.38 | 0.37 |  | 0.01 |
| 21 | Diisopentylamine | 0.16 | 0.18 | 0.15 | 0.01 |
| 22 | 1-Bromooctane | 0.21 | 0.22 | 0.22 | -0.01 |
| 23 | Vinylidenechloride | -0.45 | -0.44 |  | -0.01 |
| 24 | Vinylformate | -0.44 | -0.45 | -0.45 | 0.01 |
| 25 | Hexylcyclohexane | 0.34 | 0.35 | 0.35 | -0.01 |
| 26 | 2-Aminopentane | -0.21 | -0.20 | -0.20 | -0.01 |
| 27 | Tetrachloroethylene | -0.05 | -0.06 |  | 0.01 |
| 28 | n-Butylbenzoate | 0.49 | 0.49 | 0.50 | -0.01 |
| 29 | 2,6-Dimethylheptan-4-one | 0.01 | 0.03 | 0.02 | -0.01 |
| 30 | Tributylamine | 0.16 | 0.17 | 0.17 | -0.01 |
| 31 | N-Butylpyridinium tetrafluoroborate | 2.34 | 2.34 | 2.35 | -0.01 |
| 32 | 1-Pentanol | 0.60 | 0.60 | 0.61 | -0.01 |
| 33 | Caprylene | -0.33 | -0.32 | -0.32 | -0.01 |
| 34 | Nitrobenzene | 0.31 | 0.30 |  | 0.01 |
| 35 | Diisopropylamine | -0.38 | -0.38 | -0.39 | 0.01 |
| 36 | Ethyl methyl sulfide | -0.43 | -0.42 |  | -0.01 |
| 37 | Triethylphosphate | 0.25 | 0.26 |  | -0.01 |
| 38 | 2-Methyl-1-butyl acetate | -0.06 | -0.06 | -0.05 | -0.01 |
| 39 | 2-Methylpentane | -0.51 | -0.49 | -0.50 | -0.01 |
| 40 | 2-Butanone | -0.37 | -0.36 | -0.36 | -0.01 |
| 41 | 1-Chlorotetradecane | 0.64 | 0.65 | 0.65 | -0.01 |
| 42 | Decylcyclohexane | 0.72 | 0.71 | 0.71 | 0.01 |
| 43 | 2-Phenyloctane | 0.39 | 0.39 | 0.40 | -0.01 |
| 44 | 1-Chlorododecane | 0.47 | 0.47 | 0.46 | 0.01 |
| 45 | 1-Butanethiol | -0.30 | -0.31 | -0.31 | 0.01 |
| 46 | Propanolamine | 0.87 | 0.90 | 0.88 | -0.01 |
| 47 | Isopentene | -0.65 | -0.66 | -0.66 | 0.01 |
| 48 | Methyl methacrylate | -0.23 | -0.25 | -0.24 | 0.01 |
| 49 | 3-Methylthiophene | -0.17 | -0.16 |  | -0.01 |
| 50 | Methyl benzoate | 0.31 | 0.29 | 0.30 | 0.01 |
| 51 | 2-Fluorotoluene | -0.17 | -0.19 | -0.18 | 0.01 |
| 52 | 2-Methylbutyric acid | 0.38 | 0.37 | 0.37 | 0.01 |
| 53 | 2-Chloropropane | -0.40 | -0.41 |  | 0.01 |
| 54 | Pentanenitrile | -0.14 | -0.14 | -0.15 | 0.01 |
| 55 | Triethylamine | -0.43 | -0.37 | -0.44 | 0.01 |
| 56 | Bicyclohexyl | 0.60 | 0.62 | 0.59 | 0.01 |
| 57 | cis-2-Hexene | -0.56 | -0.54 | -0.55 | -0.01 |
| 58 | trans-2-Hexene | -0.56 | -0.54 | -0.55 | -0.01 |
| 59 | Octylcyclohexane | 0.54 | 0.53 | 0.53 | 0.01 |
| 60 | Hexylcyclopentane | 0.27 | 0.26 | 0.26 | 0.01 |
| 61 | Amyl acetate | -0.03 | -0.03 | -0.02 | -0.01 |
| 62 | 1-Chlorononane | 0.19 | 0.20 | 0.20 | -0.01 |
| 63 | n-Butylethylether | -0.38 | -0.41 | -0.39 | 0.01 |
| 64 | Vinyl acetate | -0.38 | -0.37 | -0.39 | 0.01 |
| 65 | Heptylbenzene | 0.32 | 0.33 | 0.33 | -0.01 |
| 66 | Octane | -0.27 | -0.28 | -0.28 | 0.01 |
| 67 | Trimethylethylene | -0.69 | -0.70 | -0.70 | 0.01 |
| 68 | trans-3-Hexenyl formate | -0.05 | -0.10 | -0.06 | 0.01 |
| 69 | 1-Chlorodecane | 0.29 | 0.29 | 0.30 | -0.01 |
| 70 | 4-Methylpyridine | -0.05 | -0.06 |  | 0.01 |
| 71 | Nonylcyclohexane | 0.64 | 0.62 | 0.62 | 0.02 |
| 72 | n-Hexanoic acid | 0.51 | 0.49 | 0.49 | 0.02 |
| 73 | Acetonitrile | -0.43 | -0.41 | -0.41 | -0.02 |
| 74 | Ethyl formate | -0.40 | -0.38 | -0.38 | -0.02 |
| 75 | 1,2,3,4-Tetrahydronaphthalene | 0.34 | 0.34 | 0.36 | -0.02 |
| 76 | Isobutyl acetate | -0.16 | -0.15 | -0.14 | -0.02 |
| 77 | t-Amyl mercaptan | -0.18 | -0.16 |  | -0.02 |
| 78 | Heptane | -0.39 | -0.37 | -0.37 | -0.02 |
| 79 | 1-Chlorohexadecane | 0.79 | 0.83 | 0.81 | -0.02 |
| 80 | 1-Hexanethiol | -0.11 | -0.13 | -0.13 | 0.02 |
| 81 | Undecylbenzene | 0.66 | 0.69 | 0.68 | -0.02 |
| 82 | Dibutyl phthalate | 1.30 | 1.26 | 1.28 | 0.02 |
| 83 | Methyl propyl sulfide | -0.33 | -0.33 | -0.31 | -0.02 |
| 84 | Ethyl benzoate | 0.35 | 0.31 | 0.33 | 0.02 |
| 85 | Nonylcyclopentane | 0.55 | 0.53 | 0.57 | -0.02 |
| 86 | Ethyl 2-methylbutanoate | -0.08 | -0.06 | -0.06 | -0.02 |
| 87 | 1-Methyl-4-isopropylcyclohexene | -0.03 | -0.01 | -0.01 | -0.02 |
| 88 | 1,5-Hexadiene | -0.56 | -0.54 | -0.54 | -0.02 |
| 89 | 2-Ethylhexyl benzoate | 0.83 | 0.82 | 0.85 | -0.02 |
| 90 | s-Butylbenzene | 0.02 | 0.03 | 0.04 | -0.02 |
| 91 | 1,2-Hexanediol | 1.92 | 1.87 | 1.90 | 0.02 |
| 92 | 1-Hexanol | 0.70 | 0.69 | 0.68 | 0.02 |
| 93 | Isobutyl bromide | -0.19 | -0.17 | -0.17 | -0.02 |
| 94 | Butanal | -0.34 | -0.32 | -0.32 | -0.02 |
| 95 | 1-Nonene | -0.21 | -0.23 | -0.23 | 0.02 |
| 96 | Tri-n-pentylamine | 0.44 | 0.44 | 0.42 | 0.02 |
| 97 | Heptylcyclopentane | 0.37 | 0.35 | 0.35 | 0.02 |
| 98 | 2-Propanethiol | -0.43 | -0.41 | -0.41 | -0.02 |
| 99 | 2-Methylhexane | -0.42 | -0.40 | -0.40 | -0.02 |
| 100 | 3-Ethylhexane | -0.34 | -0.31 | -0.32 | -0.02 |
| 101 | Propenal | -0.46 | -0.45 | -0.44 | -0.02 |
| 102 | 2,4-Dimethylpentane | -0.44 | -0.43 | -0.42 | -0.02 |
| 103 | Octylcyclopentane | 0.46 | 0.44 | 0.44 | 0.02 |
| 104 | n-Butyl acrylate | -0.05 | -0.07 | -0.07 | 0.02 |
| 105 | Diamylamine | 0.21 | 0.24 | 0.23 | -0.02 |
| 106 | 2-Methyl-2-butanol | 0.67 | 0.68 | 0.69 | -0.02 |
| 107 | 2-Octanamine | 0.06 | 0.07 | 0.04 | 0.02 |
| 108 | 2-Methylpropylamine | -0.22 | -0.19 | -0.19 | -0.02 |
| 109 | Pentylcyclohexane | 0.24 | 0.26 | 0.26 | -0.03 |
| 110 | 1-Hexyl-3-cyanopyridinium bis(trifluoromethylsulfonyl)amide | 3.24 | 3.22 | 3.21 | 0.03 |
| 111 | 1-Bromodecane | 0.37 | 0.40 | 0.40 | -0.03 |
| 112 | Propanal | -0.39 | -0.41 | -0.42 | 0.03 |
| 113 | Styrene | -0.12 | -0.16 | -0.15 | 0.03 |
| 114 | n-Butyl acetate | -0.14 | -0.12 | -0.11 | -0.03 |
| 115 | 1-Pentanethiol | -0.19 | -0.22 | -0.22 | 0.03 |
| 116 | Ethyl t-butyl ether | -0.38 | -0.33 | -0.35 | -0.03 |
| 117 | Benzylamine | 0.31 | 0.27 | 0.28 | 0.03 |
| 118 | 2,2,3-Trimethylpentane | -0.22 | -0.26 | -0.25 | 0.03 |
| 119 | Heptylcyclohexane | 0.45 | 0.44 | 0.42 | 0.03 |
| 120 | Benzyl acetate | 0.36 | 0.31 | 0.33 | 0.03 |
| 121 | o-Xylene | -0.09 | -0.14 | -0.12 | 0.03 |
| 122 | Thiolane | 0.02 | -0.01 | -0.01 | 0.03 |
| 123 | DEHP | 1.91 | 1.92 | 1.94 | -0.03 |
| 124 | cis-1,2-Dichloroethylene | -0.33 | -0.36 |  | 0.03 |
| 125 | Methylbenzene | -0.23 | -0.21 | -0.20 | -0.03 |
| 126 | Dimethyl sulfide | -0.54 | -0.53 | -0.51 | -0.03 |
| 127 | n-Hexylamine | -0.01 | 0.02 | 0.02 | -0.03 |
| 128 | n-Propylformate | -0.24 | -0.29 | -0.27 | 0.03 |
| 129 | Heptyl bromide | 0.10 | 0.13 | 0.13 | -0.03 |
| 130 | 3-Methylhexane | -0.43 | -0.40 | -0.40 | -0.03 |
| 131 | Tetrahydrofuran | -0.26 | -0.29 | -0.29 | 0.03 |
| 132 | Propyl isobutanoate | -0.08 | -0.06 | -0.05 | -0.03 |
| 133 | N,N-Dimethylformamide | -0.06 | -0.03 |  | -0.03 |
| 134 | Propionic anhydride | 0.06 | 0.08 | 0.09 | -0.03 |
| 135 | n-Propyl acetate | -0.23 | -0.21 | -0.20 | -0.03 |
| 136 | 1-Bromobutane | -0.19 | -0.14 | -0.16 | -0.03 |
| 137 | Di-n-butylamine | 0.02 | 0.06 | 0.05 | -0.03 |
| 138 | 3-Methylpentane | -0.52 | -0.49 | -0.49 | -0.03 |
| 139 | Ethyl acrylate | -0.27 | -0.25 | -0.24 | -0.03 |
| 140 | Dimethyl adipate | 0.53 | 0.54 | 0.56 | -0.03 |
| 141 | Dibenzylamine | 0.96 | 0.92 | 0.93 | 0.03 |
| 142 | cis-3-Hexene | -0.56 | -0.54 | -0.53 | -0.03 |
| 143 | trans-3-Hexene | -0.56 | -0.54 | -0.53 | -0.03 |
| 144 | 2-Ethylhexyl acetate | 0.18 | 0.21 | 0.21 | -0.03 |
| 145 | Methylcyclohexane | -0.13 | -0.10 | -0.10 | -0.03 |
| 146 | trans-1,2-Dichloroethylene | -0.39 | -0.36 |  | -0.03 |
| 147 | Aniline | 0.64 | 0.59 | 0.61 | 0.03 |
| 148 | Nonylbenzene | 0.50 | 0.51 | 0.53 | -0.03 |
| 149 | 2-Heptanamine | -0.04 | -0.02 | -0.01 | -0.03 |
| 150 | Diethyl adipate | 0.55 | 0.58 | 0.58 | -0.03 |
| 151 | Hexane | -0.49 | -0.46 | -0.45 | -0.04 |
| 152 | Cyclopentanone | 0.07 | 0.03 | 0.03 | 0.04 |
| 153 | Trichloroethylene | -0.25 | -0.21 |  | -0.04 |
| 154 | Fluorobenzene | -0.22 | -0.26 | -0.26 | 0.04 |
| 155 | n-Hexylacetate | 0.03 | 0.06 | 0.07 | -0.04 |
| 156 | Methyl acrylate | -0.30 | -0.27 | -0.26 | -0.04 |
| 157 | 1-Chlorooctane | 0.08 | 0.11 | 0.12 | -0.04 |
| 158 | Isobutyl iodide | -0.06 | -0.02 |  | -0.04 |
| 159 | n-Butylformate | -0.16 | -0.20 | -0.20 | 0.04 |
| 160 | 1-Octyl-3-cyanopyridinium bis(trifluoromethylsulfonyl)amide | 3.37 | 3.40 | 3.41 | -0.04 |
| 161 | Isooctane | -0.30 | -0.26 | -0.26 | -0.04 |
| 162 | cis-Decalin | 0.53 | 0.44 | 0.49 | 0.04 |
| 163 | cis-3-Hexenyl acetate | -0.05 | -0.02 | -0.01 | -0.04 |
| 164 | 2,2,4,6,6-Pentamethylheptane | 0.11 | 0.15 | 0.15 | -0.04 |
| 165 | trans-2-Octene | -0.32 | -0.36 | -0.36 | 0.04 |
| 166 | 1-Heptanethiol | -0.02 | -0.04 | -0.06 | 0.04 |
| 167 | 4-Chlorotoluene | -0.05 | -0.03 | -0.01 | -0.04 |
| 168 | Ethyl ether | -0.61 | -0.59 | -0.57 | -0.04 |
| 169 | Isopentylamine | -0.14 | -0.10 | -0.10 | -0.04 |
| 170 | Triethylmethanol | 0.83 | 0.86 | 0.87 | -0.04 |
| 171 | Isopropyl formate | -0.29 | -0.26 | -0.25 | -0.04 |
| 172 | alpha-Methylstyrene | -0.10 | -0.14 | -0.14 | 0.04 |
| 173 | 2-Butanol | 0.59 | 0.54 | 0.55 | 0.04 |
| 174 | Nonane | -0.15 | -0.19 | -0.19 | 0.04 |
| 175 | Heptadecane | 0.57 | 0.53 | 0.53 | 0.04 |
| 176 | Amylamine | -0.11 | -0.07 | -0.07 | -0.04 |
| 177 | Ethyl butanoate | -0.17 | -0.12 | -0.13 | -0.04 |
| 178 | Ethoxybenzene | 0.09 | 0.07 | 0.05 | 0.04 |
| 179 | 1,2-Dichloropropane | -0.06 | -0.02 |  | -0.04 |
| 180 | 4-Heptanone | -0.13 | -0.09 | -0.09 | -0.04 |
| 181 | 1-Octanethiol | 0.07 | 0.05 | 0.03 | 0.04 |
| 182 | Dimethylacetamide | 0.02 | 0.06 |  | -0.04 |
| 183 | Hexanenitrile | -0.02 | -0.05 | -0.06 | 0.04 |
| 184 | 1,1,2-Trichloroethane | 0.04 | 0.00 |  | 0.04 |
| 185 | 1-Chloroheptane | -0.02 | 0.02 | 0.02 | -0.04 |
| 186 | 1,3-Dichlorobenzene | 0.04 | 0.08 |  | -0.04 |
| 187 | (Chloromethyl)benzene | 0.15 | 0.18 | 0.19 | -0.04 |
| 188 | 1-Butyl-4-methylpyridinium tetrafluoroborate | 2.45 | 2.41 | 2.41 | 0.04 |
| 189 | 4-Fluorotoluene | -0.21 | -0.19 | -0.16 | -0.05 |
| 190 | Amylbenzene | 0.12 | 0.15 | 0.17 | -0.05 |
| 191 | 1,2,3-Trichloropropane | 0.41 | 0.37 | 0.36 | 0.05 |
| 192 | 2-Methylheptane | -0.28 | -0.31 | -0.33 | 0.05 |
| 193 | 2-Butanethiol | -0.29 | -0.32 | -0.34 | 0.05 |
| 194 | 1-Bromopentane | -0.09 | -0.05 | -0.04 | -0.05 |
| 195 | 1-Butyl-3-methylpyridinium tetrafluoroborate | 2.39 | 2.41 | 2.44 | -0.05 |
| 196 | Propanone | -0.50 | -0.45 | -0.45 | -0.05 |
| 197 | Isocumene | -0.07 | -0.03 | -0.02 | -0.05 |
| 198 | 2,3-Dimethylpentane | -0.39 | -0.43 | -0.44 | 0.05 |
| 199 | Diethylacetic acid | 0.52 | 0.46 | 0.47 | 0.05 |
| 200 | t-Butylmercaptan | -0.20 | -0.25 |  | 0.05 |
| 201 | 1-Ethyl-2,3-dimethylimidazolium bis(trifluoromethanesulfonyl) amide | 1.95 | 2.00 |  | -0.05 |
| 202 | 1,1,1-Trifluoro-2,3-dichloropropane | -0.06 | -0.11 |  | 0.05 |
| 203 | Acetic acid | 0.09 | 0.13 | 0.14 | -0.05 |
| 204 | 1-Bromopropane | -0.28 | -0.23 | -0.23 | -0.05 |
| 205 | Ethyl isovalerate | -0.10 | -0.06 | -0.05 | -0.05 |
| 206 | 1-Butanol | 0.47 | 0.51 | 0.52 | -0.05 |
| 207 | Pentanoic acid | 0.35 | 0.40 | 0.40 | -0.05 |
| 208 | Enanthic acid | 0.64 | 0.58 | 0.59 | 0.05 |
| 209 | Diethylketone | -0.32 | -0.27 | -0.27 | -0.05 |
| 210 | Diethyl sulphide | -0.35 | -0.31 | -0.30 | -0.05 |
| 211 | Ethyl pentanoate | -0.07 | -0.03 | -0.02 | -0.05 |
| 212 | t-Butyl acrylate | -0.04 | 0.01 | 0.01 | -0.05 |
| 213 | Benzyl propanoate | 0.37 | 0.40 | 0.42 | -0.05 |
| 214 | Cyclohexene | -0.19 | -0.24 | -0.24 | 0.05 |
| 215 | 1-Butylamine | -0.21 | -0.16 | -0.16 | -0.05 |
| 216 | Butylcyclohexane | 0.12 | 0.17 | 0.17 | -0.05 |
| 217 | 1-(2-Cyanoethyl)-3-decylimidazolium benzenesulfonate | 3.66 | 3.61 |  | 0.05 |
| 218 | 1-Chlorohexane | -0.13 | -0.07 | -0.08 | -0.05 |
| 219 | 1-Phenyldodecane | 0.74 | 0.78 | 0.79 | -0.05 |
| 220 | Dodecylcyclohexane | 0.88 | 0.89 | 0.93 | -0.05 |
| 221 | 1-Butyl-2,3-dimethylimidazolium bis(trifluoromethanesulfonyl) amide | 2.13 | 2.18 |  | -0.05 |
| 222 | Isobutyl chloride | -0.34 | -0.28 | -0.28 | -0.05 |
| 223 | Butylbenzene | 0.02 | 0.06 | 0.07 | -0.06 |
| 224 | Propyl propanoate | -0.17 | -0.12 | -0.11 | -0.06 |
| 225 | Hexyl bromide | -0.01 | 0.04 | 0.05 | -0.06 |
| 226 | 1-Heptanol | 0.85 | 0.78 | 0.79 | 0.06 |
| 227 | 1-Heptene | -0.46 | -0.41 | -0.40 | -0.06 |
| 228 | 1,1-Dichloroethane | -0.45 | -0.39 |  | -0.06 |
| 229 | 1-Decene | -0.09 | -0.14 | -0.15 | 0.06 |
| 230 | 3-Fluorotoluene | -0.22 | -0.19 | -0.16 | -0.06 |
| 231 | Acrylic acid | 0.11 | 0.18 | 0.17 | -0.06 |
| 232 | N-Propylpyridinium tetrafluoroborate | 2.20 | 2.25 | 2.26 | -0.06 |
| 233 | o-Toluidine | 0.64 | 0.66 | 0.70 | -0.06 |
| 234 | 2-Propanol | 0.39 | 0.45 | 0.45 | -0.06 |
| 235 | s-Butylamine | -0.34 | -0.29 | -0.28 | -0.06 |
| 236 | Methyl formate | -0.46 | -0.40 | -0.40 | -0.06 |
| 237 | Ethyl iodide | -0.23 | -0.17 |  | -0.06 |
| 238 | 1,2-Diaminoethane | 0.19 | 0.14 | 0.13 | 0.06 |
| 239 | 1,3-Dimethylbenzene | -0.21 | -0.14 | -0.15 | -0.06 |
| 240 | Ethylbenzene | -0.17 | -0.12 | -0.11 | -0.06 |
| 241 | Tetrahydro-2H-pyran-2-methanol | 1.04 | 1.07 | 1.10 | -0.06 |
| 242 | Propyl butyrate | -0.08 | -0.03 | -0.02 | -0.06 |
| 243 | Ethyl propanoate | -0.27 | -0.21 | -0.21 | -0.06 |
| 244 | 1-Octanol | 0.95 | 0.87 | 0.89 | 0.06 |
| 245 | n-Heptylamine | 0.16 | 0.11 | 0.10 | 0.06 |
| 246 | MTBE | -0.41 | -0.35 | -0.35 | -0.06 |
| 247 | Ethyl acetate | -0.34 | -0.30 | -0.28 | -0.06 |
| 248 | N,N-Dimethylbenzylamine | 0.12 | 0.18 | 0.18 | -0.06 |
| 249 | 1-Undecene | 0.01 | -0.05 | -0.05 | 0.06 |
| 250 | Ethylcyclohexane | -0.07 | -0.01 | -0.01 | -0.06 |
| 251 | 1-Octadecene | 0.64 | 0.58 | 0.57 | 0.06 |
| 252 | cis-2-Pentene | -0.70 | -0.63 | -0.63 | -0.06 |
| 253 | Methyl decanoate | 0.33 | 0.40 | 0.39 | -0.07 |
| 254 | Methyl-n-propylketone | -0.30 | -0.27 | -0.23 | -0.07 |
| 255 | 1-Heptadecene | 0.56 | 0.49 | 0.49 | 0.07 |
| 256 | Hexylbenzene | 0.22 | 0.24 | 0.29 | -0.07 |
| 257 | Decane | -0.03 | -0.10 | -0.10 | 0.07 |
| 258 | Isoprene | -0.65 | -0.70 | -0.72 | 0.07 |
| 259 | 2,2,4,4,6,8,8-Heptamethylnonane | 0.57 | 0.56 | 0.50 | 0.07 |
| 260 | Acetic anhydride | -0.04 | -0.10 | -0.11 | 0.07 |
| 261 | 1,4-Dimethylbenzene | -0.19 | -0.14 | -0.12 | -0.07 |
| 262 | Tridecylbenzene | 0.81 | 0.87 | 0.88 | -0.07 |
| 263 | Isopentane | -0.65 | -0.58 | -0.58 | -0.07 |
| 264 | 1-Iodopropane | -0.01 | -0.08 |  | 0.07 |
| 265 | Di-n-butylether | -0.16 | -0.23 | -0.23 | 0.07 |
| 266 | Furan | -0.42 | -0.42 | -0.49 | 0.07 |
| 267 | Decylcyclopentane | 0.55 | 0.62 | 0.62 | -0.07 |
| 268 | n-Valeraldehyde | -0.29 | -0.23 | -0.22 | -0.07 |
| 269 | Butyl decyl phthalate | 1.74 | 1.80 | 1.81 | -0.07 |
| 270 | Dibutyl maleate | 0.75 | 0.68 | 0.68 | 0.07 |
| 271 | 3,3-Dimethylhexane | -0.30 | -0.23 | -0.23 | -0.07 |
| 272 | Tridecylcyclohexane | 0.95 | 0.98 | 1.02 | -0.07 |
| 273 | Decylbenzene | 0.58 | 0.60 | 0.65 | -0.07 |
| 274 | 2-Hydroxyethylmethacrylate | 0.84 | 0.92 | 0.91 | -0.07 |
| 275 | Isobutyl formate | -0.17 | -0.23 | -0.24 | 0.07 |
| 276 | Propanenitrile | -0.37 | -0.32 | -0.30 | -0.07 |
| 277 | Trifluoroacetic acid | -0.03 | 0.04 | 0.04 | -0.07 |
| 278 | 1-Dodecene | 0.11 | 0.04 | 0.04 | 0.07 |
| 279 | 1-Hexene | -0.59 | -0.50 | -0.51 | -0.07 |
| 280 | Ethyl dodecanoate | 0.52 | 0.60 | 0.60 | -0.08 |
| 281 | 1-(2-Cyanoethyl)-3-octylimidazolium 3-sulfobenzoate | 4.37 | 4.45 |  | -0.08 |
| 282 | 1-Chloropentane | -0.24 | -0.16 | -0.16 | -0.08 |
| 283 | Methacrylic acid | 0.13 | 0.20 | 0.21 | -0.08 |
| 284 | Diethylcarbonate | -0.09 | -0.01 |  | -0.08 |
| 285 | Undecane | 0.07 | -0.01 | -0.01 | 0.08 |
| 286 | Undecylcyclopentane | 0.63 | 0.71 | 0.71 | -0.08 |
| 287 | Tetradecane | 0.34 | 0.26 | 0.26 | 0.08 |
| 288 | Diisobutyl adipate | 0.79 | 0.88 | 0.87 | -0.08 |
| 289 | Pentane | -0.62 | -0.55 | -0.54 | -0.08 |
| 290 | 2,2,2-Trifluoroethanol | 0.30 | 0.24 | 0.22 | 0.08 |
| 291 | Methyl octanoate | 0.15 | 0.22 | 0.23 | -0.08 |
| 292 | 1-Propanethiol | -0.33 | -0.40 | -0.41 | 0.08 |
| 293 | 1-Propanol | 0.35 | 0.42 | 0.43 | -0.08 |
| 294 | Bromoethane | -0.40 | -0.32 | -0.32 | -0.08 |
| 295 | 4-Methylvaleronitrile | -0.01 | -0.08 | -0.09 | 0.08 |
| 296 | 1-Tetradecene | 0.30 | 0.22 | 0.22 | 0.08 |
| 297 | 1-Pentadecene | 0.39 | 0.31 | 0.31 | 0.08 |
| 298 | Butanoic anhydride | 0.21 | 0.26 | 0.29 | -0.08 |
| 299 | 1-Tridecene | 0.21 | 0.13 | 0.13 | 0.08 |
| 300 | Cumene | -0.10 | -0.06 | -0.02 | -0.08 |
| 301 | N-Methylmorpholine | 0.01 | -0.05 | -0.07 | 0.08 |
| 302 | 3-Methyl-2-butanone | -0.24 | -0.30 | -0.32 | 0.08 |
| 303 | Dodecane | 0.17 | 0.08 | 0.09 | 0.08 |
| 304 | t-Nonylmercaptan | 0.12 | 0.20 |  | -0.08 |
| 305 | Cetane | 0.52 | 0.44 | 0.44 | 0.08 |
| 306 | Isopropylacetate | -0.25 | -0.18 | -0.16 | -0.08 |
| 307 | 4-t-Butylpyridine | 0.18 | 0.26 |  | -0.09 |
| 308 | Cetene | 0.48 | 0.40 | 0.39 | 0.09 |
| 309 | 1,2-Dibromoethane | 0.24 | 0.18 | 0.15 | 0.09 |
| 310 | Pentadecylbenzene | 0.95 | 1.05 | 1.04 | -0.09 |
| 311 | 2-Methylacrylonitrile | -0.41 | -0.34 | -0.32 | -0.09 |
| 312 | 7-Phenyltridecane | 0.94 | 0.84 | 0.85 | 0.09 |
| 313 | N-Methylformamide | 0.28 | 0.37 |  | -0.09 |
| 314 | N,N-Dimethylaniline | 0.15 | 0.09 | 0.06 | 0.09 |
| 315 | Methyllaurate | 0.50 | 0.58 | 0.59 | -0.09 |
| 316 | Benzaldehyde | 0.16 | 0.11 | 0.07 | 0.09 |
| 317 | Anisole | 0.12 | 0.05 | 0.03 | 0.09 |
| 318 | Methylenanthate | 0.05 | 0.13 | 0.14 | -0.09 |
| 319 | N-Ethyl-2-pyrrolidone | 0.33 | 0.42 |  | -0.09 |
| 320 | Methylacetoacetate | 0.23 | 0.14 | 0.14 | 0.09 |
| 321 | Methyl propanoate | -0.32 | -0.23 | -0.23 | -0.09 |
| 322 | Piperidine | 0.17 | 0.09 | 0.08 | 0.09 |
| 323 | Tripropylamine | -0.17 | -0.10 | -0.08 | -0.09 |
| 324 | Dodecylcyclopentane | 0.71 | 0.80 | 0.80 | -0.09 |
| 325 | 1,7-Dibromoheptane | 0.69 | 0.63 | 0.60 | 0.09 |
| 326 | Tridecane | 0.28 | 0.17 | 0.18 | 0.10 |
| 327 | 2,3-Dimethylbutane | -0.43 | -0.52 | -0.52 | 0.10 |
| 328 | 2-Chlorobutane | -0.39 | -0.32 | -0.29 | -0.10 |
| 329 | n-Octanoic acid | 0.77 | 0.67 | 0.67 | 0.10 |
| 330 | Butyric acid methylester | -0.24 | -0.14 | -0.14 | -0.10 |
| 331 | Ethyl acetoacetate | 0.24 | 0.16 | 0.14 | 0.10 |
| 332 | 1-Chlorobutane | -0.34 | -0.25 | -0.24 | -0.10 |
| 333 | Formamide | 0.57 | 0.47 |  | 0.10 |
| 334 | Di-n-hexylether | 0.23 | 0.13 | 0.13 | 0.10 |
| 335 | Isopropylamine | -0.46 | -0.38 | -0.36 | -0.10 |
| 336 | Benzene | -0.19 | -0.28 | -0.29 | 0.10 |
| 337 | Diisobutylamine | -0.10 | 0.00 | 0.00 | -0.10 |
| 338 | Methylcyclopentane | -0.30 | -0.19 | -0.19 | -0.10 |
| 339 | Diisoamyl ether | 0.01 | -0.11 | -0.10 | 0.11 |
| 340 | Cyanoethylene | -0.46 | -0.36 | -0.35 | -0.11 |
| 341 | Pentadecane | 0.46 | 0.35 | 0.35 | 0.11 |
| 342 | Benzyl ether | 0.73 | 0.63 | 0.62 | 0.11 |
| 343 | n-Decyl alcohol | 1.16 | 1.05 | 1.05 | 0.11 |
| 344 | Methyl hexanoate | -0.05 | 0.04 | 0.06 | -0.11 |
| 345 | Acetophenone | 0.26 | 0.16 | 0.15 | 0.11 |
| 346 | Methyl acetate | -0.42 | -0.32 | -0.31 | -0.11 |
| 347 | Tridecylcyclopentane | 0.78 | 0.89 | 0.89 | -0.11 |
| 348 | (9Z)-Octadecenoic acid | 1.59 | 1.49 | 1.48 | 0.11 |
| 349 | m-Toluidine | 0.58 | 0.66 | 0.69 | -0.11 |
| 350 | 2,2-Dimethylpentane | -0.42 | -0.32 | -0.31 | -0.11 |
| 351 | Methyl isobutanoate | -0.28 | -0.17 | -0.17 | -0.11 |
| 352 | Triptane | -0.24 | -0.35 | -0.35 | 0.11 |
| 353 | 2-Methyl-1-propanol | 0.59 | 0.48 | 0.48 | 0.11 |
| 354 | 4-Allyl-2-methoxyphenol | 0.97 | 1.04 | 1.08 | -0.12 |
| 355 | Methylvalerate | -0.15 | -0.05 | -0.03 | -0.12 |
| 356 | Cyclopentane | -0.36 | -0.25 | -0.24 | -0.12 |
| 357 | Propylcyclohexane | 0.00 | 0.08 | 0.12 | -0.12 |
| 358 | Pyridine | -0.01 | -0.13 |  | 0.12 |
| 359 | Butanoic acid | 0.19 | 0.31 | 0.31 | -0.12 |
| 360 | 1,3,5-Trimethylbenzene | 0.06 | -0.07 | -0.06 | 0.12 |
| 361 | Allyl chloride | -0.49 | -0.38 | -0.37 | -0.12 |
| 362 | Ethanethiol | -0.60 | -0.49 | -0.48 | -0.12 |
| 363 | 1-Chloropropane | -0.45 | -0.34 | -0.33 | -0.12 |
| 364 | Thietane | -0.20 | -0.10 | -0.07 | -0.12 |
| 365 | 1,2-Dichlorobenzene | 0.16 | 0.08 | 0.03 | 0.13 |
| 366 | trans-1,3,5-Trimethylcyclohexane | -0.15 | 0.02 | -0.02 | -0.13 |
| 367 | Tetradecylbenzene | 0.88 | 0.96 | 1.01 | -0.13 |
| 368 | 1-Pentene | -0.71 | -0.59 | -0.58 | -0.13 |
| 369 | 1,2-Pentanediol | 1.89 | 1.78 | 1.76 | 0.13 |
| 370 | Tetrahydropyran | -0.08 | -0.20 | -0.21 | 0.13 |
| 371 | 3-(Dimethylamino)propylamine | 0.03 | 0.14 | 0.16 | -0.13 |
| 372 | Tetradecylcyclopentane | 0.85 | 0.98 | 0.98 | -0.13 |
| 373 | 1,2,4-Trimethylbenzene | 0.05 | -0.07 | -0.08 | 0.13 |
| 374 | Ethyl myristate | 0.67 | 0.78 | 0.80 | -0.13 |
| 375 | Tri-n-heptylamine | 0.87 | 0.98 | 1.00 | -0.13 |
| 376 | 4-Ethyltoluene | -0.16 | -0.05 | -0.03 | -0.13 |
| 377 | 2-Ethyl-1-hexanol | 0.99 | 0.84 | 0.86 | 0.13 |
| 378 | Butylcyclopentane | -0.05 | 0.08 | 0.08 | -0.13 |
| 379 | Benzonitrile | 0.06 | 0.20 | 0.19 | -0.13 |
| 380 | 1-Propylamine | -0.39 | -0.25 | -0.26 | -0.13 |
| 381 | Acetaldehyde | -0.61 | -0.50 | -0.48 | -0.13 |
| 382 | Di-n-propylamine | -0.25 | -0.12 | -0.11 | -0.13 |
| 383 | Hydrocinnamyl alcohol | 1.01 | 1.12 | 1.14 | -0.14 |
| 384 | 2-Mercaptoethanol | 0.53 | 0.66 | 0.67 | -0.14 |
| 385 | Cyclopentene | -0.46 | -0.33 | -0.32 | -0.14 |
| 386 | Dodecahydrosqualene | 1.55 | 1.52 | 1.41 | 0.14 |
| 387 | Diethyl cellosolve | -0.19 | -0.36 | -0.33 | 0.14 |
| 388 | 2-Methylpropanal | -0.25 | -0.35 | -0.39 | 0.14 |
| 389 | Diisopropyl ether | -0.47 | -0.35 | -0.32 | -0.15 |
| 390 | Ethylcyclopentane | -0.25 | -0.10 | -0.10 | -0.15 |
| 391 | Ethyl vinyl ether | -0.70 | -0.57 | -0.55 | -0.15 |
| 392 | Pentylcyclopentane | 0.06 | 0.17 | 0.21 | -0.15 |
| 393 | Butyl vinyl ether | -0.30 | -0.39 | -0.45 | 0.15 |
| 394 | 1,2-Propanediol | 1.75 | 1.60 | 1.60 | 0.15 |
| 395 | Dimethyl carbonate | -0.20 | -0.05 |  | -0.15 |
| 396 | Cyclohexane | -0.01 | -0.16 | -0.16 | 0.15 |
| 397 | Pentadecylcyclopentane | 0.92 | 1.07 | 1.07 | -0.15 |
| 398 | N-Methyl-2-pyrrolidone | 0.28 | 0.39 | 0.43 | -0.15 |
| 399 | Trimethylphosphate | 0.35 | 0.20 |  | 0.15 |
| 400 | Methyloxiran | -0.49 | -0.35 | -0.33 | -0.15 |
| 401 | Ethylepoxide | -0.39 | -0.26 | -0.23 | -0.16 |
| 402 | Isopentyl alcohol | 0.71 | 0.57 | 0.55 | 0.16 |
| 403 | Propylcyclopentane | -0.17 | -0.01 | -0.01 | -0.16 |
| 404 | Bis(2-hydroxyethyl)methylamine | 2.01 | 1.90 | 1.85 | 0.16 |
| 405 | trans-Decalin | 0.33 | 0.44 | 0.49 | -0.16 |
| 406 | cis-3-Hexenyl formate | -0.22 | -0.10 | -0.06 | -0.16 |
| 407 | N-Butylaniline | 0.54 | 0.70 |  | -0.16 |
| 408 | trans-2-Pentene | -0.79 | -0.63 | -0.62 | -0.17 |
| 409 | 2-Methyltetrahydrofuran | -0.31 | -0.17 | -0.14 | -0.17 |
| 410 | Cyclohexylamine | 0.33 | 0.19 | 0.16 | 0.17 |
| 411 | 2-Methyl-1-butanol | 0.74 | 0.57 | 0.57 | 0.17 |
| 412 | Pelargonic acid | 0.92 | 0.76 | 0.75 | 0.17 |
| 413 | Triethanolamine | 2.98 | 3.08 | 3.15 | -0.17 |
| 414 | 2-Methylpropanoic acid | 0.12 | 0.28 | 0.29 | -0.17 |
| 415 | Hexadecylcyclopentane | 0.98 | 1.16 | 1.16 | -0.18 |
| 416 | Phenethyl alcohol | 0.87 | 1.03 | 1.05 | -0.18 |
| 417 | Diethylamine | -0.47 | -0.30 | -0.29 | -0.18 |
| 418 | 1-Nonanol | 1.16 | 0.96 | 0.97 | 0.19 |
| 419 | Tetrahydrodicyclopentadiene | 0.48 | 0.68 | 0.67 | -0.19 |
| 420 | Propan-1,3-diol | 1.75 | 1.57 | 1.55 | 0.20 |
| 421 | Propanoic acid | 0.04 | 0.22 | 0.24 | -0.20 |
| 422 | 2-(2-(2-Ethoxyethoxy)ethoxy)ethanol | 0.91 | 1.02 | 1.11 | -0.20 |
| 423 | Diethyleneglycol | 1.55 | 1.71 | 1.76 | -0.21 |
| 424 | 2-Methyl-2-propanol | 0.75 | 0.59 | 0.54 | 0.21 |
| 425 | 1-Butyl-3-cyanopyridinium bis(trifluoromethylsulfonyl)amide | 3.19 | 3.04 | 2.98 | 0.21 |
| 426 | 4-Ethoxyaniline | 1.11 | 0.94 | 0.90 | 0.21 |
| 427 | Propylenecarbonate | 0.44 | 0.23 |  | 0.21 |
| 428 | Tri-n-butyl phosphate | 0.59 | 0.80 |  | -0.21 |
| 429 | n-Octylamine | 0.41 | 0.20 | 0.19 | 0.21 |
| 430 | 2-Ethoxyaniline | 0.78 | 0.94 | 1.00 | -0.22 |
| 431 | 1,2-Butanediol | 1.86 | 1.69 | 1.64 | 0.22 |
| 432 | Ethan-1,2-diol | 1.30 | 1.48 | 1.52 | -0.22 |
| 433 | Benzyl alcohol | 0.75 | 0.94 | 0.97 | -0.22 |
| 434 | Bis(2-chloroethyl) ether | 0.38 | 0.19 | 0.15 | 0.23 |
| 435 | N-Methylpropionamide | 0.78 | 0.55 |  | 0.23 |
| 436 | Diethyl maleate | 0.55 | 0.32 | 0.32 | 0.23 |
| 437 | Tri-n-octylamine | 1.05 | 1.25 | 1.29 | -0.24 |
| 438 | Ethanol | 0.08 | 0.33 | 0.32 | -0.24 |
| 439 | Ethyl oleate | 0.81 | 1.06 | 1.05 | -0.24 |
| 440 | 1-Hexyl-4-cyanopyridinium bis(trifluoromethylsulfonyl)amide | 3.03 | 3.22 | 3.27 | -0.24 |
| 441 | Cyclohexanone | 0.35 | 0.12 | 0.10 | 0.25 |
| 442 | Allyl alcohol | 0.14 | 0.38 | 0.39 | -0.26 |
| 443 | Triethyleneglycol | 1.69 | 1.94 | 1.95 | -0.26 |
| 444 | Ethanediol diacetate | 0.50 | 0.22 | 0.23 | 0.27 |
| 445 | Bis(2-hydroxyethyl) sulfide | 1.81 | 1.99 | 2.08 | -0.27 |
| 446 | 2-Ethyl-1-hexanoic acid | 0.89 | 0.64 | 0.62 | 0.27 |
| 447 | 2-Ethoxyethanol | 0.31 | 0.56 | 0.58 | -0.27 |
| 448 | Acetylacetone | -0.22 | 0.01 | 0.08 | -0.30 |
| 449 | 1-(2-Cyanoethyl)-3-decylimidazolium bis(2-ethylhexyl)sulfosuccinate | 4.30 | 4.37 | 4.60 | -0.30 |
| 450 | Methylcyanoacetate | 0.45 | 0.18 | 0.14 | 0.31 |
| 451 | Diisobutyl phthalate | 1.48 | 1.20 | 1.17 | 0.31 |
| 452 | N,N-Diethylaniline | 0.34 | 0.15 | 0.02 | 0.32 |
| 453 | Methyl cellosolve | 0.24 | 0.54 | 0.56 | -0.32 |
| 454 | 1,4-Dioxane | 0.08 | -0.24 | -0.25 | 0.33 |
| 455 | Morpholine | 0.35 | 0.05 | 0.01 | 0.34 |
| 456 | Dimethylmaleate | 0.55 | 0.28 | 0.21 | 0.34 |
| 457 | 1,5-Pentanediol | 2.11 | 1.75 | 1.76 | 0.35 |
| 458 | 4-Morpholineethanol | 1.42 | 1.13 | 1.07 | 0.35 |
| 459 | Thiane | 0.39 | 0.08 | 0.03 | 0.36 |
| 460 | Dibutyl decanedioate | 0.96 | 1.30 | 1.33 | -0.37 |
