# Supplementary material for: Application of a General Computer Algorithm Based on the Group-Additivity Method for the Calculation of Two Molecular Descriptors at Both Ends of Dilution: Liquid Viscosity and Activity Coefficient in Water at Infinite Dilution
Source: Molecules. 2017 Dec 21;23(1):5. doi: 10.3390/molecules23010005 (PMC5943952; doi:10.3390/molecules23010005)
Supplement: Supplementary file 1 [file molecules-23-00005-s001.zip › S4. Experimental and Calculated Activity-Coefficient-Data Table.doc]

|  | **Molecules sorted by deviation** |  |  |  |  |
| --- | --- | --- | --- | --- | --- |
| **Entry** | **Molecule name** | **log(γ)^∞^ exp** | **log(γ)^∞^ calc** | **log(γ)^∞^ test** | **Deviation** |
| 1 | Propenal | 1.32 | 1.28 | 1.32 | 0.00 |
| 2 | Propyl butyrate | 3.65 | 3.64 | 3.65 | 0.00 |
| 3 | n-Nonylamine | 3.91 | 3.89 | 3.91 | 0.00 |
| 4 | Acenaphthylene | 5.36 | 5.36 | 5.36 | 0.00 |
| 5 | p-Cresol | 2.49 | 2.52 | 2.49 | 0.00 |
| 6 | Ethyl ether | 2.04 | 2.03 | 2.04 | 0.00 |
| 7 | Perfluoroheptane | 9.25 | 9.24 | 9.25 | 0.00 |
| 8 | 1,2,4-Trimethylbenzene | 5.07 | 5.10 | 5.07 | 0.00 |
| 9 | trans-1,2-Dimethylcyclohexane | 6.12 | 6.18 | 6.12 | 0.00 |
| 10 | 4-Heptanone | 3.22 | 3.21 | 3.22 | 0.00 |
| 11 | 1-Pentadecanol | 8.16 | 8.16 | 8.15 | 0.00 |
| 12 | Ethylamine | -0.32 | -0.31 | -0.31 | -0.01 |
| 13 | 3-Hexanone | 2.62 | 2.61 | 2.61 | 0.01 |
| 14 | 3-Methyl-2-butanol | 1.90 | 1.88 | 1.89 | 0.01 |
| 15 | Ethyl acrylate | 2.45 | 2.43 | 2.44 | 0.01 |
| 16 | Sulfamerazine | 1.78 | 1.77 |  | 0.01 |
| 17 | 1-Propylamine | 0.31 | 0.29 | 0.30 | 0.01 |
| 18 | o-Cresol | 2.44 | 2.52 | 2.45 | -0.01 |
| 19 | Ethylenedichloride | 2.81 | 2.82 | 2.80 | 0.01 |
| 20 | 3,5-Dimethyl-4-heptanol | 4.25 | 4.21 | 4.24 | 0.01 |
| 21 | 2-Chloronitrobenzene | 4.18 | 4.18 | 4.17 | 0.01 |
| 22 | n-Propylformate | 2.23 | 2.23 | 2.24 | -0.01 |
| 23 | Butylethylamine | 2.11 | 2.10 | 2.10 | 0.01 |
| 24 | Heptane | 6.28 | 6.31 | 6.29 | -0.02 |
| 25 | 3-Methyl-2-butanone | 1.92 | 1.94 | 1.94 | -0.02 |
| 26 | 3-Hexanol | 2.54 | 2.55 | 2.56 | -0.02 |
| 27 | Diethylketone | 2.03 | 2.01 | 2.01 | 0.02 |
| 28 | 1,2-Dibromobenzene | 5.24 | 5.24 | 5.26 | -0.02 |
| 29 | Ethanethiol | 2.37 | 2.30 | 2.35 | 0.02 |
| 30 | 3-Pentanol | 1.92 | 1.95 | 1.94 | -0.02 |
| 31 | 2-Chlorophenol | 2.68 | 2.73 | 2.70 | -0.02 |
| 32 | Cryofluorane | 4.84 | 4.86 |  | -0.02 |
| 33 | n-Nonanal | 4.88 | 4.89 | 4.86 | 0.02 |
| 34 | 2-Methyl-2-hexanol | 2.82 | 2.79 | 2.80 | 0.02 |
| 35 | 1-Nitrobutane | 3.09 | 3.11 |  | -0.02 |
| 36 | n-Hexylamine | 2.13 | 2.09 | 2.15 | -0.02 |
| 37 | trans-1,2-Dichloroethylene | 3.10 | 3.08 | 3.08 | 0.02 |
| 38 | o-Xylene | 4.48 | 4.58 | 4.50 | -0.02 |
| 39 | Heptabarbital | 3.00 | 3.02 | 3.02 | -0.02 |
| 40 | 1-Bromopropane | 3.46 | 3.40 | 3.44 | 0.02 |
| 41 | 2-Methyltetrahydrofuran | 1.61 | 1.64 | 1.63 | -0.02 |
| 42 | 1,2,4-Tribromobenzene | 6.20 | 6.18 | 6.18 | 0.02 |
| 43 | Pentachlorobenzene | 6.79 | 6.86 | 6.81 | -0.02 |
| 44 | 2,4-Dimethylpentane | 6.12 | 6.17 | 6.14 | -0.02 |
| 45 | 3-Ethylpyridine | 2.30 | 2.30 | 2.28 | 0.02 |
| 46 | 1,3-Dimethylbenzene | 4.52 | 4.58 | 4.50 | 0.02 |
| 47 | 1-Iodobutane | 4.69 | 4.64 | 4.67 | 0.02 |
| 48 | 4-Nitrophenol | 2.16 | 2.15 | 2.18 | -0.02 |
| 49 | 2-Undecanone | 5.62 | 5.61 | 5.60 | 0.02 |
| 50 | 4-Ethoxyacetanilide | 2.32 | 2.32 | 2.30 | 0.02 |
| 51 | Methyl isoamyl ketone | 3.18 | 3.14 | 3.16 | 0.02 |
| 52 | 2-Picoline | 1.57 | 1.55 | 1.55 | 0.02 |
| 53 | 2,3-Dimethyl-2-butanol | 2.12 | 2.12 | 2.14 | -0.02 |
| 54 | 4-Methylacetophenone | 3.16 | 3.17 | 3.14 | 0.02 |
| 55 | Cyclohexylamine | 1.73 | 1.71 | 1.75 | -0.02 |
| 56 | 2-Methyl-3-pentanol | 2.44 | 2.48 | 2.47 | -0.03 |
| 57 | 2-Pentanol | 1.99 | 1.95 | 1.96 | 0.03 |
| 58 | Ethylisopropylketone | 2.56 | 2.54 | 2.53 | 0.03 |
| 59 | 2,6-Lutidine | 1.90 | 1.89 | 1.87 | 0.03 |
| 60 | Butylcellosolve | 1.58 | 1.61 | 1.55 | 0.03 |
| 61 | 2,4-Dinitrotoluene | 4.10 | 4.09 | 4.07 | 0.03 |
| 62 | Bromobenzene | 4.35 | 4.30 | 4.32 | 0.03 |
| 63 | Ethyl formate | 1.67 | 1.63 | 1.64 | 0.03 |
| 64 | 2-Ethylpyridine | 2.17 | 2.15 | 2.14 | 0.03 |
| 65 | Di-n-propylamine | 2.13 | 2.10 | 2.10 | 0.03 |
| 66 | Isobutyl acetate | 2.93 | 2.97 | 2.96 | -0.03 |
| 67 | Vinyl acetate | 2.26 | 2.30 | 2.29 | -0.03 |
| 68 | Isobutylbenzene | 5.87 | 5.88 | 5.84 | 0.03 |
| 69 | 2,6-Dimethyl-4-heptanol | 4.25 | 4.21 | 4.22 | 0.03 |
| 70 | 4-Nonanol | 4.33 | 4.35 | 4.36 | -0.03 |
| 71 | 2,3-Lutidine | 2.04 | 2.04 | 2.01 | 0.03 |
| 72 | 1,1,1-Trichloroethane | 3.77 | 3.75 | 3.74 | 0.03 |
| 73 | 1,2,4-Trichlorobenzene | 5.46 | 5.46 | 5.43 | 0.03 |
| 74 | 2-Octanone | 3.77 | 3.81 | 3.80 | -0.03 |
| 75 | Amylobarbitone | 2.52 | 2.50 | 2.49 | 0.03 |
| 76 | 2,2-Dimethyl-3-pentanol | 2.89 | 2.87 | 2.86 | 0.03 |
| 77 | 3-Buten-2-one | 1.38 | 1.40 | 1.41 | -0.03 |
| 78 | 1-Nitropropane | 2.48 | 2.51 |  | -0.03 |
| 79 | 2,5-Lutidine | 2.05 | 2.04 | 2.01 | 0.04 |
| 80 | 1,4-Difluorobenzene | 3.72 | 3.68 | 3.68 | 0.04 |
| 81 | Octylbenzene | 8.30 | 8.47 | 8.34 | -0.04 |
| 82 | Propanone | 0.85 | 0.81 | 0.81 | 0.04 |
| 83 | 2,4-Dichlorophenol | 3.40 | 3.43 | 3.44 | -0.04 |
| 84 | 4-Methyloctane | 7.80 | 7.82 | 7.76 | 0.04 |
| 85 | Benzylamine | 1.53 | 1.56 | 1.57 | -0.04 |
| 86 | Isobutyronitrile | 2.07 | 2.05 | 2.03 | 0.04 |
| 87 | Methyl-n-propylketone | 1.97 | 2.01 | 2.01 | -0.04 |
| 88 | 2,3-Dimethyl-1-butene | 4.52 | 4.51 | 4.48 | 0.04 |
| 89 | 3-Nonanol | 4.40 | 4.35 | 4.36 | 0.04 |
| 90 | Nitroethane | 1.95 | 1.91 |  | 0.04 |
| 91 | 1,2,3-Trichlorobenzene | 5.42 | 5.46 | 5.46 | -0.04 |
| 92 | Methylbenzene | 3.96 | 4.06 | 4.00 | -0.04 |
| 93 | 3-Methyl-3-hexanol | 2.73 | 2.79 | 2.77 | -0.04 |
| 94 | 1,3-Dimethylnaphthalene | 6.04 | 6.14 | 6.08 | -0.04 |
| 95 | 2-Methyl-2-butanol | 1.55 | 1.59 | 1.59 | -0.04 |
| 96 | Ethyl acetate | 1.81 | 1.84 | 1.85 | -0.04 |
| 97 | Ethyl trans-cinnamate | 4.83 | 4.91 | 4.87 | -0.04 |
| 98 | N-(4-Chlorophenyl)benzenesulfonamide | 4.67 | 4.79 | 4.63 | 0.04 |
| 99 | 4-Chlorobenzoic acid | 2.68 | 2.72 | 2.72 | -0.04 |
| 100 | 2,3-Dimethylbutane | 5.36 | 5.38 | 5.40 | -0.04 |
| 101 | 1-Bromo-4-chlorobenzene | 4.98 | 5.00 | 5.02 | -0.04 |
| 102 | 3-Methyl-2-pentanol | 2.46 | 2.48 | 2.50 | -0.04 |
| 103 | Di-n-propyl phthalate | 5.18 | 5.26 | 5.22 | -0.04 |
| 104 | Diisopropyl ether | 2.80 | 2.81 | 2.84 | -0.04 |
| 105 | Isopropylacetate | 2.29 | 2.23 | 2.25 | 0.04 |
| 106 | Ethyl butanoate | 3.00 | 3.04 | 3.04 | -0.04 |
| 107 | 2-Butanol | 1.32 | 1.35 | 1.36 | -0.04 |
| 108 | 3-Nonanone | 4.38 | 4.41 | 4.42 | -0.04 |
| 109 | 2-Decanone | 5.04 | 5.01 | 5.00 | 0.04 |
| 110 | Refrigerant 115 | 4.29 | 4.25 |  | 0.04 |
| 111 | Dimethyl phthalate | 3.38 | 3.38 | 3.34 | 0.04 |
| 112 | Methyl methoxyacetate | 1.17 | 1.21 | 1.21 | -0.04 |
| 113 | Allyl chloride | 3.03 | 2.99 | 2.98 | 0.05 |
| 114 | Methyl isobutyl ketone | 2.47 | 2.54 | 2.51 | -0.05 |
| 115 | 1,3-Difluorobenzene | 3.75 | 3.68 | 3.70 | 0.05 |
| 116 | 4-Methylpyridine | 1.64 | 1.70 | 1.69 | -0.05 |
| 117 | Propyl propanoate | 3.09 | 3.04 | 3.04 | 0.05 |
| 118 | 4-Amino-N-(2-bromo-4-nitrophenyl)benzenesulfonamide | 4.07 | 4.10 | 4.12 | -0.05 |
| 119 | Bromoethane | 2.83 | 2.80 | 2.78 | 0.05 |
| 120 | trans-1,4-Dimethylcyclohexane | 6.21 | 6.18 | 6.16 | 0.05 |
| 121 | Ethyl propanoate | 2.41 | 2.44 | 2.46 | -0.05 |
| 122 | n-Octylamine | 3.34 | 3.29 | 3.29 | 0.05 |
| 123 | Ethyl t-amyl ether | 3.30 | 3.26 | 3.25 | 0.05 |
| 124 | 1,2,3,5-Tetrafluorobenzene | 4.05 | 4.00 | 4.00 | 0.05 |
| 125 | 3,3-Dimethylpentane | 5.97 | 6.03 | 6.02 | -0.05 |
| 126 | Isobutyl formate | 2.79 | 2.76 | 2.74 | 0.05 |
| 127 | Perphenazine | 3.95 | 3.90 |  | 0.05 |
| 128 | Prehnitene | 5.63 | 5.62 | 5.58 | 0.05 |
| 129 | 4-Amino-N-(4-chloro-2-methylphenyl)-benzenesulfonamide | 4.21 | 4.23 | 4.16 | 0.05 |
| 130 | 4-Amino-N-(3,4-dichlorophenyl)benzene sulfonamide | 4.52 | 4.44 | 4.47 | 0.05 |
| 131 | Methylchloroacetate | 1.98 | 1.92 | 1.93 | 0.05 |
| 132 | Acetonitrile | 0.97 | 0.92 | 0.91 | 0.05 |
| 133 | 2,4-Dimethyl-3-pentanone | 3.05 | 3.07 | 3.10 | -0.06 |
| 134 | 3-Heptanone | 3.15 | 3.21 | 3.20 | -0.06 |
| 135 | 2,4-Xylenol | 2.96 | 3.01 | 3.01 | -0.06 |
| 136 | n-Propyl acetate | 2.38 | 2.44 | 2.44 | -0.06 |
| 137 | 2,6-Xylenol | 2.95 | 3.01 | 3.01 | -0.06 |
| 138 | 2-Methyl-2-propanol | 1.06 | 0.99 | 1.00 | 0.06 |
| 139 | 2-Hexanone | 2.55 | 2.61 | 2.61 | -0.06 |
| 140 | Myristyl alcohol | 7.60 | 7.56 | 7.54 | 0.06 |
| 141 | 1,3,5-Trimethylbenzene | 5.07 | 5.10 | 5.13 | -0.06 |
| 142 | 1,3-Dichloropropane | 3.36 | 3.42 | 3.42 | -0.06 |
| 143 | 2,2-Dimethylpentane | 6.10 | 6.03 | 6.04 | 0.06 |
| 144 | 1,4-Dimethylbenzene | 4.52 | 4.58 | 4.58 | -0.06 |
| 145 | Ethyl t-butyl ether | 2.71 | 2.66 | 2.65 | 0.06 |
| 146 | Undecane | 9.39 | 9.47 | 9.45 | -0.06 |
| 147 | Sulfadimidine | 2.05 | 2.11 |  | -0.06 |
| 148 | N-(3-Chloro-2-pyridinyl)-benzenesulfonamide | 3.64 | 3.58 |  | 0.06 |
| 149 | 3-Methylpyridine | 1.74 | 1.70 | 1.68 | 0.06 |
| 150 | Di-n-butylether | 4.50 | 4.43 | 4.44 | 0.06 |
| 151 | 2,5-Dimethyltetrahydrofuran | 2.07 | 2.03 | 2.01 | 0.06 |
| 152 | Methyl cellosolve | -0.07 | 0.07 | 0.00 | -0.07 |
| 153 | Amylamine | 1.47 | 1.49 | 1.54 | -0.07 |
| 154 | 1-(1-Methylethoxy)propane | 3.08 | 3.02 | 3.01 | 0.07 |
| 155 | 3,5-Lutidine | 2.24 | 2.19 | 2.17 | 0.07 |
| 156 | Isopropyl butanoate | 3.49 | 3.43 | 3.42 | 0.07 |
| 157 | 1,2,3,4-Tetrachlorobenzene | 6.11 | 6.16 | 6.18 | -0.07 |
| 158 | Iodobenzene | 4.73 | 4.68 | 4.66 | 0.07 |
| 159 | n-Heptaldehyde | 3.62 | 3.69 | 3.69 | -0.07 |
| 160 | 4-Aminophenol | 0.92 | 0.98 | 0.99 | -0.07 |
| 161 | 1,1,2-Trichloroethane | 3.18 | 3.20 | 3.25 | -0.07 |
| 162 | 4-Chloroaniline | 2.94 | 3.01 | 3.01 | -0.07 |
| 163 | 1-Methylnaphthalene | 5.45 | 5.62 | 5.52 | -0.07 |
| 164 | Butyronitrile | 2.05 | 2.12 | 2.12 | -0.07 |
| 165 | Methyllactate | 0.30 | 0.27 | 0.23 | 0.07 |
| 166 | Methyl benzoate | 3.28 | 3.37 | 3.35 | -0.07 |
| 167 | 4-Methyl-2-pentanol | 2.54 | 2.48 | 2.47 | 0.07 |
| 168 | 2,3-Dimethyl-2-pentanol | 2.62 | 2.72 | 2.69 | -0.07 |
| 169 | 2-Heptanone | 3.15 | 3.21 | 3.22 | -0.07 |
| 170 | 1-Propanethiol | 3.18 | 2.90 | 3.10 | 0.07 |
| 171 | Allyl alcohol | 0.88 | 0.95 | 0.95 | -0.08 |
| 172 | 2-Butanone | 1.33 | 1.41 | 1.41 | -0.08 |
| 173 | 3-Chloroiodobenzene | 5.29 | 5.38 | 5.37 | -0.08 |
| 174 | 1-Propoxy-2-propanol | 1.50 | 1.40 | 1.42 | 0.08 |
| 175 | 1,2-Dichloropropane | 3.37 | 3.42 | 3.45 | -0.08 |
| 176 | 1-Bromobutane | 4.09 | 4.00 | 4.01 | 0.08 |
| 177 | 1-Nitronaphthalene | 4.97 | 4.92 | 4.89 | 0.08 |
| 178 | (Chloromethyl)benzene | 4.16 | 4.27 | 4.24 | -0.08 |
| 179 | N-(4-Nitrophenyl)benzenesulfonamide | 4.29 | 4.21 | 4.21 | 0.08 |
| 180 | Formic acid | -0.14 | -0.06 | -0.06 | -0.08 |
| 181 | 2,4-Lutidine | 1.96 | 2.04 | 2.04 | -0.08 |
| 182 | 2-Methyl-1-butanol | 2.17 | 2.09 | 2.09 | 0.08 |
| 183 | Methyl nonanoate | 5.62 | 5.70 | 5.70 | -0.09 |
| 184 | n-Butylformate | 2.75 | 2.83 | 2.84 | -0.09 |
| 185 | 1,4-Pentadiene | 3.83 | 3.91 | 3.92 | -0.09 |
| 186 | m-Cresol | 2.44 | 2.52 | 2.53 | -0.09 |
| 187 | 1,2-Dichlorobenzene | 4.83 | 4.76 | 4.74 | 0.09 |
| 188 | 2-Bromobutane | 3.92 | 3.83 |  | 0.09 |
| 189 | alpha-Estradiol | 4.42 | 4.35 | 4.33 | 0.09 |
| 190 | 2-Bromopropane | 3.32 | 3.23 |  | 0.09 |
| 191 | 2-Hexanol | 2.45 | 2.55 | 2.54 | -0.09 |
| 192 | Acetaldehyde | 0.60 | 0.69 | 0.69 | -0.09 |
| 193 | 2-Methylacrylonitrile | 2.22 | 2.14 | 2.13 | 0.09 |
| 194 | 1,2,4,5-Tetrachlorobenzene | 6.07 | 6.16 | 6.16 | -0.09 |
| 195 | Cyclohexanone | 1.93 | 1.83 | 1.84 | 0.09 |
| 196 | 3-Methyl-3-pentanol | 2.11 | 2.19 | 2.20 | -0.09 |
| 197 | 2-Chloroiodobenzene | 5.29 | 5.38 | 5.38 | -0.09 |
| 198 | Ethylbenzene | 4.52 | 4.69 | 4.61 | -0.10 |
| 199 | Pyrene | 7.04 | 7.04 | 6.94 | 0.10 |
| 200 | 3,3-Dimethyl-2-butanol | 2.36 | 2.27 | 2.26 | 0.10 |
| 201 | 3,3-Dimethyl-1-butanol | 2.36 | 2.48 | 2.46 | -0.10 |
| 202 | 1,1,2,2-Tetrachloroethane | 3.54 | 3.58 | 3.64 | -0.10 |
| 203 | Chlorobenzene | 4.15 | 4.06 | 4.05 | 0.10 |
| 204 | 1,3-Dichlorobenzene | 4.82 | 4.76 | 4.72 | 0.10 |
| 205 | 1-Iodohexane | 5.92 | 5.84 | 5.82 | 0.10 |
| 206 | Nonylbenzene | 8.99 | 9.10 | 9.09 | -0.10 |
| 207 | 5-Nonanone | 4.34 | 4.41 | 4.44 | -0.10 |
| 208 | 2,3,5,6-Tetrachloroanisole | 6.30 | 6.18 | 6.19 | 0.11 |
| 209 | 1,2-Difluorobenzene | 3.75 | 3.68 | 3.64 | 0.11 |
| 210 | 2,4,6-Trinitrotoluene | 4.29 | 4.21 | 4.18 | 0.11 |
| 211 | 1,6-Heptadiene | 5.08 | 5.17 | 5.19 | -0.11 |
| 212 | Benzo[e]pyrene | 8.41 | 8.60 | 8.52 | -0.11 |
| 213 | Xylocaine | 3.12 | 3.12 | 3.01 | 0.11 |
| 214 | Methylcyclohexane | 5.56 | 5.46 | 5.45 | 0.11 |
| 215 | 5-Nonanol | 4.23 | 4.35 | 4.34 | -0.11 |
| 216 | 2-Chlorobutane | 3.71 | 3.60 |  | 0.11 |
| 217 | Ethyl nonanoate | 5.93 | 6.04 | 6.04 | -0.11 |
| 218 | Isoamyl acetate | 3.47 | 3.57 | 3.58 | -0.11 |
| 219 | 1,2,4,5-Tetrabromobenzene | 7.05 | 7.12 | 7.16 | -0.11 |
| 220 | Ethyl iodide | 3.34 | 3.44 | 3.45 | -0.11 |
| 221 | 3,4-Lutidine | 2.10 | 2.19 | 2.21 | -0.11 |
| 222 | Propanenitrile | 1.42 | 1.52 | 1.53 | -0.11 |
| 223 | Cyanoethylene | 1.61 | 1.51 | 1.50 | 0.11 |
| 224 | 4-Ethylpyridine | 2.20 | 2.30 | 2.31 | -0.11 |
| 225 | 1-Chloronaphthalene | 5.59 | 5.50 | 5.48 | 0.11 |
| 226 | Nitrobenzene | 3.53 | 3.48 | 3.42 | 0.11 |
| 227 | 1,2,3,5-Tetrachlorobenzene | 6.28 | 6.16 | 6.16 | 0.12 |
| 228 | n-Heptylamine | 2.80 | 2.69 | 2.68 | 0.12 |
| 229 | Tetrahydropyran | 1.90 | 1.85 | 1.78 | 0.12 |
| 230 | 2-Chloronaphthalene | 5.62 | 5.50 | 5.50 | 0.12 |
| 231 | Methyl formate | 1.19 | 1.29 | 1.31 | -0.12 |
| 232 | Benzophenone | 4.63 | 4.55 | 4.51 | 0.12 |
| 233 | 1,4-Dibromobenzene | 5.34 | 5.24 | 5.22 | 0.12 |
| 234 | 2,3-Dimethylpentane | 6.03 | 6.17 | 6.15 | -0.12 |
| 235 | N-(5-Methyl-2-pyridinyl)-4-nitrobenzenesulfonamide | 3.37 | 3.49 |  | -0.12 |
| 236 | 2,2,3-Trimethyl-3-pentanol | 3.02 | 3.11 | 3.14 | -0.12 |
| 237 | Dipropyl ether | 3.36 | 3.23 | 3.24 | 0.12 |
| 238 | Pyrrolidine | 0.20 | 0.12 | 0.08 | 0.12 |
| 239 | Isopentyl alcohol | 2.20 | 2.09 | 2.07 | 0.13 |
| 240 | Metoxuron | 2.56 | 2.67 | 2.69 | -0.13 |
| 241 | Pentanenitrile | 2.60 | 2.72 | 2.73 | -0.13 |
| 242 | Furfural | 1.93 | 1.71 | 1.80 | 0.13 |
| 243 | Pentobarbital | 2.39 | 2.50 | 2.52 | -0.13 |
| 244 | Aniline | 2.18 | 2.31 | 2.31 | -0.13 |
| 245 | Diethyl phthalate | 4.13 | 4.06 | 4.00 | 0.13 |
| 246 | Ethylisopropylether | 2.30 | 2.42 | 2.43 | -0.13 |
| 247 | Crotonaldehyde | 1.84 | 1.89 | 1.97 | -0.13 |
| 248 | 2-Methylhexane | 6.34 | 6.24 | 6.21 | 0.13 |
| 249 | 3-Chloronitrobenzene | 4.30 | 4.18 | 4.17 | 0.13 |
| 250 | 1-Butanol | 1.68 | 1.56 | 1.55 | 0.13 |
| 251 | 1-Iodoheptane | 6.55 | 6.44 | 6.42 | 0.13 |
| 252 | Cyclogeraniolane | 6.60 | 6.76 | 6.73 | -0.13 |
| 253 | 1-Pentyne | 3.39 | 3.25 | 3.26 | 0.13 |
| 254 | Cumene | 5.13 | 5.25 | 5.26 | -0.13 |
| 255 | 2,3,4-Trichloroanisole | 5.60 | 5.48 | 5.46 | 0.14 |
| 256 | Allobarbital | 2.06 | 1.95 | 1.92 | 0.14 |
| 257 | 4-Amino-N-(4-chlorophenyl)-2-methyl-benzenesulfonamide | 4.10 | 4.23 | 4.23 | -0.14 |
| 258 | 1,3-Dibromobenzene | 5.12 | 5.24 | 5.26 | -0.14 |
| 259 | 1-Pentanol | 2.30 | 2.16 | 2.16 | 0.14 |
| 260 | 2-Nitrotoluene | 4.07 | 3.97 | 3.93 | 0.14 |
| 261 | Isopentyl formate | 3.26 | 3.36 | 3.40 | -0.14 |
| 262 | 4-Vinylcyclohexene | 5.08 | 4.96 | 4.94 | 0.14 |
| 263 | n-Butyl acetate | 2.91 | 3.04 | 3.05 | -0.14 |
| 264 | 2-Methylpentane | 5.54 | 5.45 | 5.40 | 0.14 |
| 265 | N-Methyl-2-pyrrolidone | 0.61 | 0.67 | 0.75 | -0.14 |
| 266 | 1,2,4,5-Tetrafluorobenzene | 4.12 | 4.00 | 3.98 | 0.14 |
| 267 | 2-Nonanol | 4.48 | 4.35 | 4.34 | 0.14 |
| 268 | 1-Iodopropane | 3.93 | 4.04 | 4.07 | -0.14 |
| 269 | Amyl acetate | 3.51 | 3.64 | 3.65 | -0.14 |
| 270 | 2-Propanol | 0.88 | 0.75 | 0.74 | 0.14 |
| 271 | 2,5-Dimethylfuran | 3.73 | 3.65 | 3.59 | 0.14 |
| 272 | 1,4-Cyclohexadiene | 3.68 | 3.78 | 3.82 | -0.14 |
| 273 | 4-Chloroacetanilide | 2.78 | 2.66 | 2.64 | 0.14 |
| 274 | 3-Methyl-2-pentanone | 2.42 | 2.54 | 2.56 | -0.14 |
| 275 | N-(2-Chlorophenyl)benzenesulfonamide | 4.59 | 4.79 | 4.73 | -0.14 |
| 276 | 2-Methylnaphthalene | 5.46 | 5.62 | 5.60 | -0.15 |
| 277 | Hexane | 5.63 | 5.52 | 5.48 | 0.15 |
| 278 | Benzene | 3.39 | 3.54 | 3.54 | -0.15 |
| 279 | Methyl decanoate | 6.43 | 6.30 | 6.28 | 0.15 |
| 280 | 1,5-Hexadiene | 4.43 | 4.54 | 4.58 | -0.15 |
| 281 | 3-Nitrophenol | 2.24 | 2.15 | 2.09 | 0.15 |
| 282 | Hexachlorobenzene | 7.71 | 7.56 | 7.56 | 0.15 |
| 283 | Allyl bromide | 3.25 | 3.39 | 3.40 | -0.15 |
| 284 | Isoprene | 3.77 | 3.94 | 3.92 | -0.15 |
| 285 | Codeine | 1.98 | 1.84 | 1.83 | 0.15 |
| 286 | Methyl acetate | 1.35 | 1.50 | 1.50 | -0.15 |
| 287 | 3-Methylpentane | 5.57 | 5.45 | 5.42 | 0.15 |
| 288 | 2-Chlorobiphenyl | 6.22 | 6.42 | 6.37 | -0.15 |
| 289 | Methyl isobutanoate | 2.49 | 2.63 | 2.64 | -0.15 |
| 290 | 1-Butyne | 2.74 | 2.62 | 2.59 | 0.15 |
| 291 | 2,3-Dimethyl-3-pentanol | 2.59 | 2.72 | 2.74 | -0.15 |
| 292 | Ethyl hexanoate | 4.10 | 4.24 | 4.25 | -0.15 |
| 293 | n-Hexylacetate | 4.10 | 4.24 | 4.25 | -0.15 |
| 294 | Isoproturon | 3.46 | 3.57 | 3.61 | -0.15 |
| 295 | Pentanoic acid | 2.10 | 1.95 | 1.95 | 0.15 |
| 296 | Pyridine | 1.33 | 1.21 | 1.18 | 0.15 |
| 297 | 1-Hexadecanol | 8.59 | 8.76 | 8.74 | -0.16 |
| 298 | 1,2-Diaminobenzene | 1.40 | 1.26 | 1.24 | 0.16 |
| 299 | Isonicotinamide | 0.61 | 0.64 | 0.77 | -0.16 |
| 300 | Pinacolone | 2.46 | 2.33 | 2.30 | 0.16 |
| 301 | Cyclohexane | 4.90 | 4.74 | 4.74 | 0.16 |
| 302 | 1,2-Dibromopropane | 3.89 | 4.05 |  | -0.16 |
| 303 | Methyl propanoate | 1.94 | 2.10 | 2.10 | -0.16 |
| 304 | Ethyl pentanoate | 3.46 | 3.64 | 3.62 | -0.16 |
| 305 | Fluorobenzene | 3.68 | 3.52 | 3.52 | 0.16 |
| 306 | cis-1,2-Dimethylcyclohexane | 6.02 | 6.18 | 6.18 | -0.16 |
| 307 | 1-Hexanol | 2.90 | 2.76 | 2.74 | 0.16 |
| 308 | n-Butyl methyl ether | 2.74 | 2.89 | 2.90 | -0.16 |
| 309 | N-Phenyl benzenesulfonamide | 4.16 | 4.09 | 3.99 | 0.17 |
| 310 | N-Methylpiperidine | 1.59 | 1.63 | 1.76 | -0.17 |
| 311 | n-Valeraldehyde | 2.34 | 2.49 | 2.51 | -0.17 |
| 312 | 4-Chloronitrobenzene | 4.33 | 4.18 | 4.16 | 0.17 |
| 313 | 1-Octanol | 4.15 | 3.96 | 3.98 | 0.17 |
| 314 | 3,5-Dichlorophenol | 3.26 | 3.43 | 3.43 | -0.17 |
| 315 | 2-Ethylnaphthalene | 6.04 | 6.25 | 6.21 | -0.17 |
| 316 | 1-Heptanol | 3.52 | 3.36 | 3.34 | 0.18 |
| 317 | Isocumene | 5.13 | 5.32 | 5.31 | -0.18 |
| 318 | 1-Octadecanol | 10.14 | 9.96 | 9.96 | 0.18 |
| 319 | 2-Methyl-1-propanol | 1.65 | 1.49 | 1.47 | 0.18 |
| 320 | Diethylamine | 0.73 | 0.90 | 0.91 | -0.18 |
| 321 | Butylbenzene | 5.71 | 5.95 | 5.89 | -0.18 |
| 322 | Isobutyl chloride | 3.75 | 3.53 | 3.57 | 0.18 |
| 323 | Acetic acid | -0.04 | 0.15 | 0.14 | -0.18 |
| 324 | Hemimellitene | 4.95 | 5.10 | 5.13 | -0.18 |
| 325 | 4-Bromoacetanilide | 3.08 | 2.90 | 2.90 | 0.18 |
| 326 | Octane | 6.98 | 7.10 | 7.16 | -0.18 |
| 327 | 1-Methylpyrrole | 2.50 | 2.32 |  | 0.18 |
| 328 | Hexanenitrile | 3.15 | 3.32 | 3.33 | -0.18 |
| 329 | Isobutylmethylether | 2.65 | 2.82 | 2.83 | -0.19 |
| 330 | 2-Furoic acid | 1.10 | 1.17 | 1.29 | -0.19 |
| 331 | 1-Propanol | 1.13 | 0.96 | 0.94 | 0.19 |
| 332 | 2-Methylfuran | 3.18 | 3.00 | 2.99 | 0.19 |
| 333 | Propyne | 2.17 | 1.99 | 1.98 | 0.19 |
| 334 | Nonane | 7.68 | 7.89 | 7.87 | -0.19 |
| 335 | Methanol | 0.20 | 0.02 | 0.01 | 0.19 |
| 336 | Methyl octanoate | 4.91 | 5.10 | 5.10 | -0.19 |
| 337 | Cyclohexylacetate | 3.43 | 3.25 | 3.24 | 0.19 |
| 338 | Diisobutyl phthalate | 6.15 | 6.32 | 6.34 | -0.19 |
| 339 | Propanal | 1.11 | 1.29 | 1.30 | -0.19 |
| 340 | 2-Methyl-4-penten-3-ol | 2.25 | 2.47 | 2.44 | -0.19 |
| 341 | 2-Chlorotoluene | 4.72 | 4.55 | 4.53 | 0.19 |
| 342 | 4-Bromotoluene | 4.95 | 4.79 | 4.76 | 0.19 |
| 343 | 3-Bromobenzoic acid | 3.10 | 2.96 | 2.91 | 0.19 |
| 344 | Hexylbenzene | 6.97 | 7.21 | 7.16 | -0.20 |
| 345 | Isopropyl propionate | 3.04 | 2.83 | 2.84 | 0.20 |
| 346 | s-trans-1,3-Butadiene | 3.14 | 3.28 | 3.34 | -0.20 |
| 347 | Methylpropylether | 2.12 | 2.29 | 2.32 | -0.20 |
| 348 | Neohexane | 5.36 | 5.24 | 5.16 | 0.20 |
| 349 | Caproic aldehyde | 2.91 | 3.09 | 3.11 | -0.20 |
| 350 | 1,4-Dimethylnaphthalene | 5.88 | 6.14 | 6.08 | -0.20 |
| 351 | s-Butylmethylether | 2.48 | 2.68 | 2.68 | -0.20 |
| 352 | Propylene | 2.82 | 2.66 | 2.62 | 0.20 |
| 353 | 4-Chlorotoluene | 4.74 | 4.55 | 4.54 | 0.20 |
| 354 | Butanal | 1.69 | 1.89 | 1.89 | -0.20 |
| 355 | Pentachloroethane | 4.41 | 4.55 | 4.61 | -0.20 |
| 356 | 1-Heptyne | 4.75 | 4.51 | 4.55 | 0.20 |
| 357 | 1,2,3-Trichloropropane | 3.64 | 3.84 |  | -0.20 |
| 358 | 1-Bromo-2-chloroethane | 3.07 | 3.22 | 3.27 | -0.21 |
| 359 | 4-Amino-2-methyl-N-(2-nitrophenyl)-benzenesulfonamide | 3.91 | 3.79 | 3.70 | 0.21 |
| 360 | 3,5-Xylenol | 2.78 | 3.01 | 2.99 | -0.21 |
| 361 | Diethyl cellosolve | 1.89 | 2.08 | 2.10 | -0.21 |
| 362 | Chloroethane | 2.60 | 2.40 | 2.39 | 0.21 |
| 363 | Methacrolein | 1.79 | 1.91 | 2.00 | -0.21 |
| 364 | Guajen | 5.87 | 6.14 | 6.08 | -0.21 |
| 365 | Ethyl-n-propylether | 2.41 | 2.63 | 2.62 | -0.21 |
| 366 | Phenanthrene | 6.41 | 6.66 | 6.62 | -0.21 |
| 367 | Isobutylene | 3.51 | 3.32 | 3.30 | 0.21 |
| 368 | 3-Nitrotoluene | 4.17 | 3.97 | 3.96 | 0.21 |
| 369 | Ethanol | 0.57 | 0.36 | 0.36 | 0.21 |
| 370 | 2,2,4-Trimethyl-4-methoxypentane | 4.59 | 4.44 | 4.38 | 0.21 |
| 371 | o-Toluidine | 2.57 | 2.80 | 2.78 | -0.22 |
| 372 | Methyl methacrylate | 2.58 | 2.72 | 2.80 | -0.22 |
| 373 | 2-Methyl-1-pentene | 4.78 | 4.58 | 4.56 | 0.22 |
| 374 | 1-Nonanol | 4.79 | 4.56 | 4.57 | 0.22 |
| 375 | t-Butylbenzene | 5.40 | 5.67 | 5.62 | -0.22 |
| 376 | Triethylmethanol | 2.58 | 2.79 | 2.80 | -0.22 |
| 377 | 1,2,4,5-Tetramethylbenzene | 5.78 | 5.62 | 5.56 | 0.22 |
| 378 | Amylbenzene | 6.36 | 6.58 | 6.58 | -0.22 |
| 379 | Isobutyl bromide | 4.15 | 3.93 | 3.93 | 0.22 |
| 380 | 1-Hexen-3-ol | 2.34 | 2.54 | 2.56 | -0.23 |
| 381 | n-Decyl alcohol | 5.38 | 5.16 | 5.15 | 0.23 |
| 382 | N-(2,3-Dichlorophenyl)benzenesulfonamide | 5.29 | 5.49 | 5.52 | -0.23 |
| 383 | n-Hexanoic acid | 2.78 | 2.55 | 2.55 | 0.23 |
| 384 | Methylvalerate | 3.10 | 3.30 | 3.33 | -0.23 |
| 385 | 1-Bromopentane | 4.82 | 4.60 | 4.59 | 0.23 |
| 386 | 4-Amino-N-(4-ethylphenyl)benzenesulfonamide | 4.28 | 4.13 | 4.05 | 0.23 |
| 387 | Bis(2-chloroethyl) ether | 2.74 | 2.87 | 2.97 | -0.23 |
| 388 | s-Butylbenzene | 5.63 | 5.88 | 5.86 | -0.23 |
| 389 | 2-Nonanone | 4.21 | 4.41 | 4.44 | -0.23 |
| 390 | 4-Nitrotoluene | 4.19 | 3.97 | 3.96 | 0.23 |
| 391 | Perfluorohexane | 8.31 | 8.12 | 8.08 | 0.23 |
| 392 | Neopentyl alcohol | 2.13 | 1.88 | 1.90 | 0.23 |
| 393 | 1-Undecanol | 5.99 | 5.76 | 5.75 | 0.24 |
| 394 | MTBE | 2.14 | 2.32 | 2.38 | -0.24 |
| 395 | Phenylacetonitrile | 3.17 | 3.39 | 3.41 | -0.24 |
| 396 | 2,3,5-Trimethylphenol | 3.27 | 3.50 | 3.51 | -0.24 |
| 397 | 1-Hexyne | 4.09 | 3.88 | 3.85 | 0.24 |
| 398 | 1-Penten-3-ol | 1.72 | 1.94 | 1.96 | -0.24 |
| 399 | 4-Methoxybenzaldehyde | 2.76 | 2.58 | 2.52 | 0.24 |
| 400 | Chloroacetic acid ethylester | 2.51 | 2.26 | 2.27 | 0.24 |
| 401 | 1,2-Dibromoethane | 3.40 | 3.62 | 3.64 | -0.24 |
| 402 | cis-1,2-Dichloroethylene | 2.94 | 3.08 | 3.18 | -0.24 |
| 403 | 1-Heptadecanol | 9.25 | 9.36 | 9.49 | -0.24 |
| 404 | Amyl propionate | 4.00 | 4.24 | 4.24 | -0.25 |
| 405 | 1-Octyne | 5.41 | 5.14 | 5.16 | 0.25 |
| 406 | Dibutyl phthalate | 6.19 | 6.46 | 6.44 | -0.25 |
| 407 | 1-Chloropropane | 3.24 | 3.00 | 2.99 | 0.25 |
| 408 | Ethyl benzoate | 3.94 | 3.71 | 3.69 | 0.25 |
| 409 | 2-Chloropropane | 3.17 | 3.00 | 2.92 | 0.25 |
| 410 | Naphthalene | 4.83 | 5.10 | 5.08 | -0.25 |
| 411 | 2-Naphthol | 3.22 | 3.47 | 3.47 | -0.25 |
| 412 | Piperidine | 0.94 | 0.72 | 0.69 | 0.25 |
| 413 | Anisole | 3.56 | 3.38 | 3.31 | 0.25 |
| 414 | Phenol | 1.79 | 2.03 | 2.04 | -0.26 |
| 415 | 1-Dodecanol | 6.65 | 6.36 | 6.39 | 0.26 |
| 416 | Methyl t-pentyl ether | 2.72 | 2.92 | 2.98 | -0.26 |
| 417 | 2-Ethyltoluene | 4.95 | 5.21 | 5.21 | -0.26 |
| 418 | N-Ethylaniline | 3.38 | 3.30 | 3.12 | 0.26 |
| 419 | 1,1,3-Trimethylcyclopentane | 6.22 | 5.97 | 5.96 | 0.26 |
| 420 | Imipramine | 4.19 | 4.25 | 4.45 | -0.26 |
| 421 | Pentane | 4.98 | 4.73 | 4.71 | 0.27 |
| 422 | 2-Ethoxyethylacetate | 1.63 | 1.89 | 1.90 | -0.27 |
| 423 | 2,5-Xylenol | 2.67 | 3.01 | 2.94 | -0.27 |
| 424 | 1,4-Dichlorobenzene | 5.01 | 4.76 | 4.74 | 0.27 |
| 425 | 4-Penten-1-ol | 1.89 | 2.15 | 2.16 | -0.27 |
| 426 | Isopropyl methyl ether | 1.80 | 2.08 | 2.07 | -0.27 |
| 427 | 2-Methoxyethyl acetate | 1.29 | 1.55 | 1.56 | -0.27 |
| 428 | Butyric acid methylester | 2.43 | 2.70 | 2.70 | -0.27 |
| 429 | Ephedrine | 2.05 | 2.19 | 2.33 | -0.28 |
| 430 | Isopentane | 4.92 | 4.66 | 4.64 | 0.28 |
| 431 | 1-Nonyne | 5.98 | 5.77 | 5.70 | 0.28 |
| 432 | Hexafluorobenzene | 4.22 | 4.32 | 4.50 | -0.28 |
| 433 | Tetrahydrofuran | 1.54 | 1.25 | 1.26 | 0.28 |
| 434 | 4-Amino-N-(2-chloro-4-methylphenyl)-benzenesulfonamide | 4.42 | 4.23 | 4.14 | 0.28 |
| 435 | 2,4,6-Trichlorophenol | 3.88 | 4.13 | 4.16 | -0.29 |
| 436 | 1-Heptene | 5.48 | 5.18 | 5.19 | 0.29 |
| 437 | 1,1-Dichloroethane | 3.03 | 2.78 | 2.74 | 0.29 |
| 438 | 3-Penten-2-ol | 1.68 | 1.95 | 1.97 | -0.29 |
| 439 | o-Anisic acid | 2.44 | 2.18 | 2.15 | 0.29 |
| 440 | 1-Chlorobutane | 3.88 | 3.60 | 3.59 | 0.29 |
| 441 | 1-Ethylnaphthalene | 5.91 | 6.25 | 6.20 | -0.29 |
| 442 | Perfluoropentane | 7.14 | 7.00 | 6.85 | 0.29 |
| 443 | trans-2-Pentene | 4.28 | 3.93 | 3.99 | 0.29 |
| 444 | Propylcyclopentane | 6.49 | 6.25 | 6.19 | 0.29 |
| 445 | 1-Nonene | 6.80 | 6.44 | 6.50 | 0.30 |
| 446 | Aprobarbital | 1.71 | 1.89 | 2.01 | -0.30 |
| 447 | 1-Chloroheptane | 5.74 | 5.40 | 5.44 | 0.30 |
| 448 | Diethyl malonate | 2.60 | 2.30 | 2.30 | 0.30 |
| 449 | 2,6-Dinitrotoluene | 4.37 | 4.09 | 4.07 | 0.30 |
| 450 | 3-Bromophenol | 3.30 | 2.97 | 3.00 | 0.30 |
| 451 | 1-Naphthol | 3.19 | 3.47 | 3.49 | -0.30 |
| 452 | Aspirin | 2.00 | 2.17 | 2.30 | -0.30 |
| 453 | Ethyl decanoate | 6.34 | 6.64 | 6.64 | -0.30 |
| 454 | 1,1,1,2-Tetrachloroethane | 3.93 | 4.17 | 4.23 | -0.30 |
| 455 | 4-Methylstyrene | 4.87 | 5.20 | 5.17 | -0.30 |
| 456 | 4-Chlorophenol | 2.44 | 2.73 | 2.74 | -0.30 |
| 457 | 1,1-Dimethyl-3-phenylurea | 1.66 | 1.95 | 1.97 | -0.31 |
| 458 | Trimethylethylene | 4.25 | 3.96 | 3.94 | 0.31 |
| 459 | 2-Bromophenol | 2.70 | 2.97 | 3.01 | -0.31 |
| 460 | Cyclooctane | 6.01 | 6.32 | 6.32 | -0.31 |
| 461 | 1-Butanethiol | 3.79 | 3.50 | 3.47 | 0.31 |
| 462 | Cyclopentanone | 1.51 | 1.23 | 1.19 | 0.32 |
| 463 | 3-Methylstyrene | 4.87 | 5.20 | 5.19 | -0.32 |
| 464 | 3,4-Xylenol | 2.70 | 3.01 | 3.02 | -0.33 |
| 465 | 1-Pentadecene | 10.51 | 10.22 | 10.18 | 0.33 |
| 466 | 4-Nitrobenzoic acid | 1.84 | 2.14 | 2.17 | -0.33 |
| 467 | Methyl hexanoate | 3.60 | 3.90 | 3.93 | -0.33 |
| 468 | 2-Chlorobenzoic acid | 2.37 | 2.72 | 2.70 | -0.33 |
| 469 | 4-s-Butylphenol | 3.90 | 4.25 | 4.23 | -0.33 |
| 470 | 4-Amino-N-(2,3-dichlorophenyl)benzene sulfonamide | 4.03 | 4.44 | 4.36 | -0.33 |
| 471 | 2,2-Dimethylpropane | 4.74 | 4.45 | 4.40 | 0.34 |
| 472 | 1-Chlorohexane | 5.15 | 4.80 | 4.81 | 0.34 |
| 473 | 1,2-Dinitrobenzene | 3.24 | 3.60 | 3.58 | -0.34 |
| 474 | Acetophenone | 2.99 | 2.68 | 2.65 | 0.34 |
| 475 | Cycloheptane | 5.26 | 5.53 | 5.60 | -0.34 |
| 476 | Heptyl bromide | 6.17 | 5.80 | 5.83 | 0.34 |
| 477 | 4-Fluoroacetanilide | 1.78 | 2.12 | 2.12 | -0.34 |
| 478 | 2,2,2-Trifluoroethanol | 0.94 | 1.19 | 1.28 | -0.34 |
| 479 | 2,6-Dimethylheptan-4-one | 3.94 | 4.27 | 4.29 | -0.35 |
| 480 | Ethene | 2.29 | 2.02 | 1.94 | 0.35 |
| 481 | Acenaphthene | 5.67 | 5.36 | 5.32 | 0.35 |
| 482 | Methylamine | -0.76 | -0.66 | -0.41 | -0.35 |
| 483 | Isopropyl isobutanoate | 3.68 | 3.36 | 3.32 | 0.36 |
| 484 | Styrene | 4.26 | 4.68 | 4.62 | -0.36 |
| 485 | 3-Nitrobenzoic acid | 2.47 | 2.14 | 2.11 | 0.36 |
| 486 | Ethyl heptanoate | 4.48 | 4.84 | 4.84 | -0.36 |
| 487 | Veratrole | 3.06 | 3.40 | 3.42 | -0.36 |
| 488 | Pentachlorophenol | 5.17 | 5.53 | 5.53 | -0.36 |
| 489 | Phenethyl alcohol | 2.44 | 2.83 | 2.80 | -0.36 |
| 490 | 4-Amino-N-(5-chloro-2-methylphenyl)-benzenesulfonamide | 3.89 | 4.23 | 4.26 | -0.37 |
| 491 | 4-Amino-2-chloro-N-(4-chlorophenyl)-benzenesulfonamide | 4.10 | 4.44 | 4.47 | -0.37 |
| 492 | Hexyl bromide | 5.55 | 5.20 | 5.18 | 0.37 |
| 493 | trans-2-Heptene | 5.56 | 5.19 | 5.19 | 0.37 |
| 494 | Indane | 4.78 | 4.43 | 4.41 | 0.37 |
| 495 | 2,6-Dimethylnaphthalene | 5.65 | 6.14 | 6.02 | -0.37 |
| 496 | 4-Ethyltoluene | 4.85 | 5.21 | 5.22 | -0.37 |
| 497 | 4-Nitro-N-(4-ethoxyphenyl)-benzenesulfonamide | 4.22 | 4.57 | 4.59 | -0.37 |
| 498 | t-Pentylbenzene | 5.90 | 6.30 | 6.27 | -0.37 |
| 499 | 4-Methylbenzaldehyde | 3.40 | 3.05 | 3.02 | 0.38 |
| 500 | Ethyl caprylate | 5.13 | 5.44 | 5.51 | -0.38 |
| 501 | Caprylic aldehyde | 3.92 | 4.29 | 4.30 | -0.38 |
| 502 | Methylcyclopentane | 5.04 | 4.67 | 4.66 | 0.38 |
| 503 | Cyclohexanol | 2.14 | 1.77 | 1.76 | 0.38 |
| 504 | 1,4-Dihydroxybenzene | 0.38 | 0.70 | 0.76 | -0.38 |
| 505 | 3-Chlorobenzoic acid | 3.07 | 2.72 | 2.69 | 0.38 |
| 506 | Di-n-hexylether | 7.16 | 6.83 | 6.78 | 0.38 |
| 507 | Fluorene | 6.02 | 5.71 | 5.63 | 0.39 |
| 508 | Benzyl benzoate | 5.87 | 5.58 | 5.48 | 0.39 |
| 509 | 2-(2,4-Dichlorophenoxy)propanoic acid | 3.32 | 3.68 | 3.71 | -0.39 |
| 510 | Phenylacetate | 3.12 | 3.37 | 3.51 | -0.39 |
| 511 | Isopropyl formate | 2.38 | 2.02 | 1.98 | 0.40 |
| 512 | Biphenyl | 5.67 | 6.08 | 6.06 | -0.40 |
| 513 | 4-Amino-N-(3-chloro-4-methylphenyl)benzenesulfonamide | 3.81 | 4.23 | 4.21 | -0.40 |
| 514 | Phenylacetic acid | 2.25 | 2.62 | 2.65 | -0.40 |
| 515 | 3,5-Dinitrobenzoic acid | 1.90 | 2.26 | 2.30 | -0.40 |
| 516 | cis- Cyclooctene | 5.42 | 5.04 | 5.02 | 0.40 |
| 517 | Ethyl t-octyl ether | 5.15 | 4.78 | 4.75 | 0.40 |
| 518 | 2,4,6-Trinitrophenol | 2.20 | 2.53 | 2.61 | -0.41 |
| 519 | 4-Carboxyphenol | 1.04 | 0.69 | 0.63 | 0.41 |
| 520 | 5-Methyl-2-furaldehyde | 2.11 | 2.36 | 2.52 | -0.41 |
| 521 | Anthracene | 6.11 | 6.66 | 6.52 | -0.41 |
| 522 | 2-Ethylaniline | 2.99 | 3.40 | 3.40 | -0.41 |
| 523 | trans-1,3-Dichloropropene | 3.15 | 3.55 | 3.56 | -0.41 |
| 524 | Butane | 4.32 | 3.94 | 3.90 | 0.42 |
| 525 | 1,6-Heptadiyne | 3.49 | 3.83 | 3.91 | -0.42 |
| 526 | 2,2-Dimethyl-1-butanol | 2.87 | 2.48 | 2.45 | 0.42 |
| 527 | 1,3,5-Trichlorobenzene | 5.85 | 5.46 | 5.43 | 0.42 |
| 528 | Triethylamine | 1.83 | 2.16 | 2.25 | -0.42 |
| 529 | Benz[a]anthracene | 7.73 | 8.22 | 8.16 | -0.43 |
| 530 | 2-Hexen-4-ol | 2.14 | 2.55 | 2.57 | -0.43 |
| 531 | 1-Butene | 3.68 | 3.29 | 3.25 | 0.43 |
| 532 | 1-Bromooctane | 6.81 | 6.40 | 6.38 | 0.43 |
| 533 | 1,8-Nonadiyne | 4.72 | 5.09 | 5.15 | -0.43 |
| 534 | Benzaldehyde | 2.96 | 2.56 | 2.52 | 0.44 |
| 535 | 1-Hexene | 4.95 | 4.55 | 4.51 | 0.44 |
| 536 | 3-Ethoxyphenol | 2.74 | 2.39 | 2.30 | 0.44 |
| 537 | Dimethyl sulfide | 2.25 | 2.69 |  | -0.44 |
| 538 | Ethyne | 1.46 | 1.44 | 1.90 | -0.45 |
| 539 | Pyrrole | 1.88 | 2.05 | 2.33 | -0.45 |
| 540 | Furan | 2.58 | 2.35 | 2.13 | 0.45 |
| 541 | 4-t-Butylphenol | 3.64 | 4.04 | 4.09 | -0.45 |
| 542 | 1,3-Dinitrobenzene | 3.15 | 3.60 | 3.60 | -0.45 |
| 543 | Decane | 8.20 | 8.68 | 8.66 | -0.46 |
| 544 | 2-Methylpropane | 4.30 | 3.87 | 3.84 | 0.46 |
| 545 | Methylacetoacetate | 1.37 | 0.93 | 0.91 | 0.46 |
| 546 | 3,4-Dimethoxybenzoic acid | 2.47 | 2.06 | 2.00 | 0.47 |
| 547 | Ethoxybenzene | 4.20 | 3.72 | 3.73 | 0.47 |
| 548 | Hexachloroethane | 5.71 | 5.52 | 5.24 | 0.47 |
| 549 | N-(4-Methoxyphenyl)sulfanilamide | 3.43 | 3.06 | 2.96 | 0.47 |
| 550 | 4-Amino-N-(4-iodophenyl)benzenesulfonamide | 3.94 | 4.36 | 4.42 | -0.48 |
| 551 | Phenothiazine | 5.15 | 5.22 | 5.63 | -0.48 |
| 552 | Fluoranthene | 7.02 | 6.64 | 6.54 | 0.48 |
| 553 | t-Butylacetate | 2.94 | 2.47 | 2.46 | 0.48 |
| 554 | Quinalbarbitone | 2.73 | 3.09 | 3.21 | -0.48 |
| 555 | Benzonitrile | 3.19 | 2.79 | 2.71 | 0.48 |
| 556 | Cyclopropane | 2.83 | 2.37 | 2.34 | 0.49 |
| 557 | 2-Isopropyl-5-methylphenol | 3.65 | 4.14 | 4.14 | -0.49 |
| 558 | 4-Methylbenzoic acid | 2.98 | 2.51 | 2.49 | 0.49 |
| 559 | Perylene | 7.98 | 8.60 | 8.48 | -0.50 |
| 560 | 2-Naphthoic acid | 3.90 | 3.46 | 3.40 | 0.50 |
| 561 | Benzoic acid | 2.51 | 2.02 | 2.01 | 0.50 |
| 562 | Cyclobarbital | 2.02 | 2.42 | 2.52 | -0.50 |
| 563 | Cyclopentane | 4.40 | 3.95 | 3.90 | 0.50 |
| 564 | 1,4-Diiodobenzene | 6.22 | 6.00 | 5.72 | 0.50 |
| 565 | 1,3-Dibromopropane | 3.82 | 4.22 | 4.32 | -0.50 |
| 566 | N-Methylpyrrolidine | 1.49 | 1.03 | 0.98 | 0.51 |
| 567 | 4-Methyl-1-pentene | 4.99 | 4.48 | 4.48 | 0.51 |
| 568 | 3-Methylbenzaldehyde | 3.55 | 3.05 | 3.04 | 0.51 |
| 569 | Cyclopentanol | 1.71 | 1.17 | 1.19 | 0.52 |
| 570 | 1,3-Dihydroxybenzene | 0.20 | 0.70 | 0.72 | -0.52 |
| 571 | Hexabromobenzene | 9.28 | 9.00 | 8.76 | 0.52 |
| 572 | Methanethiol | 2.04 | 2.32 | 2.57 | -0.53 |
| 573 | Benzo[a]pyrene | 9.05 | 8.60 | 8.52 | 0.53 |
| 574 | 1-Pentene | 4.42 | 3.92 | 3.89 | 0.53 |
| 575 | 4-Bromophenol | 2.47 | 2.97 | 3.00 | -0.53 |
| 576 | Butabarbital | 2.28 | 1.90 | 1.75 | 0.53 |
| 577 | Perfluoro-n-octane | 10.16 | 10.36 | 10.70 | -0.54 |
| 578 | 2-Aminophenol | 0.47 | 0.98 | 1.01 | -0.54 |
| 579 | Caprylene | 6.36 | 5.81 | 5.81 | 0.55 |
| 580 | Butyl pentanoate | 4.29 | 4.84 | 4.84 | -0.55 |
| 581 | 1-Methylcyclohexene | 5.01 | 4.44 | 4.46 | 0.55 |
| 582 | Propane | 3.68 | 3.15 | 3.13 | 0.55 |
| 583 | 1-Ethylpiperidine | 2.45 | 1.98 | 1.89 | 0.56 |
| 584 | Diethyl sulphide | 3.21 | 2.65 |  | 0.56 |
| 585 | Benzo[ghi]perylene | 9.35 | 8.98 | 8.78 | 0.57 |
| 586 | 2-Methylbenzoic acid | 3.06 | 2.51 | 2.49 | 0.57 |
| 587 | 2-Methoxyphenol | 2.53 | 2.05 | 1.96 | 0.57 |
| 588 | Cycloheptene | 4.91 | 4.41 | 4.34 | 0.57 |
| 589 | Pentamethylbenzene | 5.57 | 6.14 | 6.14 | -0.57 |
| 590 | Ethyl acetoacetate | 1.84 | 1.27 | 1.26 | 0.57 |
| 591 | 1,4-Dinitrobenzene | 3.00 | 3.60 | 3.58 | -0.59 |
| 592 | 4-Amino-N-(2-chlorophenyl)-2-methyl-benzenesulfonamide | 3.67 | 4.23 | 4.26 | -0.59 |
| 593 | Acetanilide | 1.38 | 1.96 | 1.98 | -0.60 |
| 594 | Diphenyl ether | 5.69 | 5.25 | 5.09 | 0.60 |
| 595 | 1,2-Dihydroxybenzene | 1.30 | 0.70 | 0.68 | 0.61 |
| 596 | 4-Nitroaniline | 3.03 | 2.43 | 2.41 | 0.62 |
| 597 | 3-Methyl-1-butene | 4.48 | 3.85 | 3.85 | 0.63 |
| 598 | Tripropylamine | 3.42 | 3.96 | 4.05 | -0.63 |
| 599 | Chloroethylene | 3.11 | 2.52 | 2.48 | 0.63 |
| 600 | Butyl 4-aminobenzoate | 4.46 | 3.86 | 3.82 | 0.64 |
| 601 | Diuron | 3.82 | 3.35 | 3.17 | 0.65 |
| 602 | Allyl ether | 2.61 | 3.21 | 3.26 | -0.65 |
| 603 | 4-Amino-N-(2,5-dichlorophenyl)benzenesulfonamide | 3.74 | 4.44 | 4.40 | -0.66 |
| 604 | trans-4-Hexen-1-ol | 2.15 | 2.76 | 2.81 | -0.66 |
| 605 | 2,6-Dichlorophenol | 4.08 | 3.43 | 3.42 | 0.66 |
| 606 | 11-Deoxycorticosterone | 3.82 | 4.29 | 4.49 | -0.67 |
| 607 | Testosterone | 4.19 | 4.72 | 4.86 | -0.67 |
| 608 | Ethane-1,2-dithiol | 2.36 | 2.62 | 3.04 | -0.68 |
| 609 | 1-Naphthalenecarboxylic acid | 4.07 | 3.46 | 3.39 | 0.68 |
| 610 | Cyclohexene | 4.45 | 3.78 | 3.76 | 0.69 |
| 611 | 1,3,5-Cycloheptatriene | 3.90 | 4.41 | 4.59 | -0.69 |
| 612 | 4-Nitroacetanilide | 2.69 | 2.08 | 1.99 | 0.70 |
| 613 | Paracetamol | 1.23 | 0.63 | 0.52 | 0.71 |
| 614 | Oxirane | 0.79 | 0.05 | 0.06 | 0.73 |
| 615 | 2-Nitrobenzoic acid | 1.69 | 2.28 | 2.42 | -0.73 |
| 616 | Vinyl ether | 2.70 | 3.11 | 3.44 | -0.74 |
| 617 | Cyclopentene | 3.85 | 3.15 | 3.11 | 0.74 |
| 618 | N-(2,4-Dibromophenyl)benzenesulfonamide | 5.31 | 5.97 | 6.05 | -0.74 |
| 619 | Ethane | 3.09 | 2.36 | 2.34 | 0.75 |
| 620 | 4-Amino-N-(4-chlorophenyl)benzene sulfonamide | 4.41 | 3.74 | 3.65 | 0.76 |
| 621 | 1,4-Dioxane | 0.73 | 0.10 | -0.04 | 0.77 |
| 622 | 4-Methoxybenzoic acid | 2.79 | 2.04 | 2.02 | 0.77 |
| 623 | Di-n-octyl phthalate | 10.62 | 11.26 | 11.40 | -0.78 |
| 624 | 4-Amino-N-(4-nitrophenyl)benzenesulfonamide | 3.87 | 3.16 | 3.09 | 0.78 |
| 625 | 3-Methylbenzoic acid | 3.28 | 2.51 | 2.49 | 0.79 |
| 626 | Pentylcyclohexane | 7.82 | 8.62 | 8.62 | -0.80 |
| 627 | 4-Amino-N-(3-chloro-2-methoxyphenyl)benzenesulfonamide | 4.49 | 3.76 | 3.69 | 0.80 |
| 628 | 1,2,3,5-Tetramethylbenzene | 6.33 | 5.62 | 5.52 | 0.81 |
| 629 | trans-2-Methyldecalin | 8.32 | 7.70 | 7.51 | 0.81 |
| 630 | Barbital | 1.42 | 0.77 | 0.60 | 0.82 |
| 631 | 4-Methoxyacetanilide | 1.30 | 1.98 | 2.12 | -0.82 |
| 632 | Corticosterone | 2.98 | 2.46 | 2.16 | 0.82 |
| 633 | N-(2-Nitrophenyl)benzenesulfonamide | 4.98 | 4.35 | 4.08 | 0.90 |
| 634 | Dodecane | 9.42 | 10.26 | 10.36 | -0.94 |
